# Neutrophils—From Bone Marrow to First-Line Defense of the Innate Immune System

**DOI:** 10.3389/fimmu.2021.767175

**Published:** 2021-12-23

**Authors:** Richard Felix Kraus, Michael Andreas Gruber

**Affiliations:** Department of Anesthesiology, University Medical Center Regensburg, Regensburg, Germany

**Keywords:** microtubule organization center, NEtosis, tumor association, neutrophil (PMN) function, neutrophil extravasation, extracellular matrix (ECM), chemotactic gradients, bidirectional (trans)migration

## Abstract

Neutrophils (polymorphonuclear cells; PMNs) form a first line of defense against pathogens and are therefore an important component of the innate immune response. As a result of poorly controlled activation, however, PMNs can also mediate tissue damage in numerous diseases, often by increasing tissue inflammation and injury. According to current knowledge, PMNs are not only part of the pathogenesis of infectious and autoimmune diseases but also of conditions with disturbed tissue homeostasis such as trauma and shock. Scientific advances in the past two decades have changed the role of neutrophils from that of solely immune defense cells to cells that are responsible for the general integrity of the body, even in the absence of pathogens. To better understand PMN function in the human organism, our review outlines the role of PMNs within the innate immune system. This review provides an overview of the migration of PMNs from the vascular compartment to the target tissue as well as their chemotactic processes and illuminates crucial neutrophil immune properties at the site of the lesion. The review is focused on the formation of chemotactic gradients in interaction with the extracellular matrix (ECM) and the influence of the ECM on PMN function. In addition, our review summarizes current knowledge about the phenomenon of bidirectional and reverse PMN migration, neutrophil microtubules, and the microtubule organizing center in PMN migration. As a conclusive feature, we review and discuss new findings about neutrophil behavior in cancer environment and tumor tissue.

## 1 The Role of Neutrophils in Non-Specific Immune Defense

Granulocytes are an important component of the innate immune system. The three types of granulocytes eosinophils, basophils, and neutrophils are distinguished by their histological, morphological, and immunological properties. Each of the three types matures in the bone marrow (1). The view that PMNs only have a life span of a few hours to fewer than 3 days after maturation has recently been challenged. Crucial aspects of the neutrophil life cycle, namely their life span in different tissues and different inflammatory states, are still considered not yet fully defined (1, 2).

**Figure 1:**
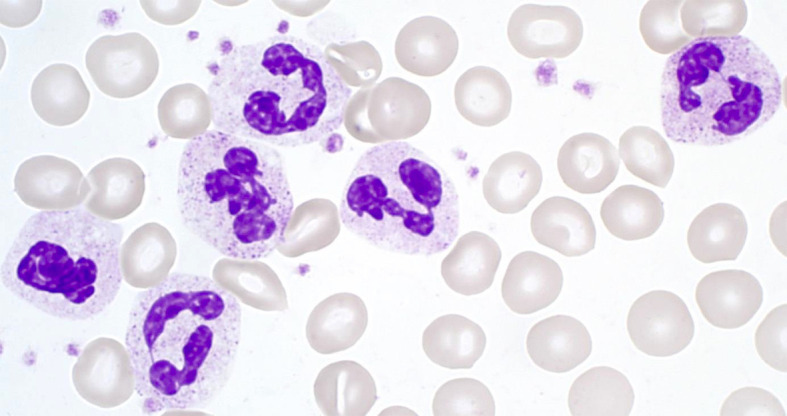
Segmented neutrophil granulocytes in Pappenheim‐stained blood cell smears (graphic provided by the laboratory for Paediatric Oncology and Haematology at the University Medical Centre Regensburg).

Making up 50–70% of all circulating leukocytes, neutrophil granulocytes (neutrophils, polymorphonuclear cells; PMNs) are the most mobile and abundant cellular component of the innate immune system of the human body. They act as an important first line of defense within the innate immune response (see below) (3, 4). Neutrophils have a diameter of 10–12 µm, and their nucleus is usually lobed into three to four segments. Therefore, PMNs are also referred to as being polymorphonuclear. Their granules are very small (<1 µm) and have a pinkish to lilac color when exposed to Pappenheim staining (see **Figure 1**) (5).

**Figure 2:**
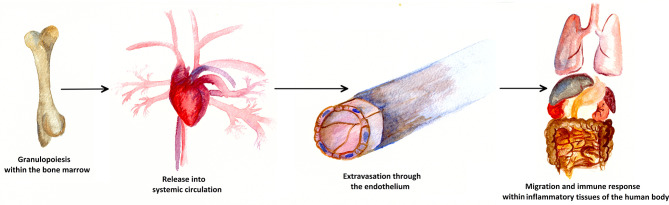
Life cycle of a neutrophil cell. Approximately 10^11^ PMNs are generated in the bone marrow *via* granulopoiesis every day. Attracted by cytokines, PMNs are consecutively released into the blood stream and thus into systemic circulation. At the sites of inflammation, PMNs leave the blood vessels through the endothelium, a process known as extravasation. In inflammatory human tissue, PMNs migrate along chemotactic gradients in the interstitium and perform specific neutrophil immune functions as a first line defense of the innate immune system.

The neutrophil life cycle begins with the granulopoiesis in the bone marrow and is illustrated in **Figure 2**. Every day, approximately 10^11^ PMNs are generated in the hematopoietic strands interspersed in the venous sinuses of the bone marrow in the human body. Granulocyte differentiation is regulated by the coordinated expression of myeloid key transcription factors (6, 7). The amount of PMNs released and renewed daily constitutes about 1% of all nucleated cells (approximately 10^13^) of the human body (8, 9). If PMNs did not have any crucial role within the human immune system, such an enormous effort would probably have become obsolete long ago in the history of evolution.

To leave the bone marrow, mature neutrophils have to migrate through the sinusoidal endothelium separating the hematopoietic compartment from the blood stream. Thereby, neutrophils seem to migrate across the bone marrow endothelium through tight-fitting pores in the transcellular rather than the paracellular pathway (7, 10, 11). For this process, endothelial penetrability is of importance, whereby endothelial homoeostasis and, as a consequence, cell release from the bone marrow are regulated and decisively influenced by vascular endothelial cadherin (12).

PMN release is stimulated by gradients across the sinus wall of bone marrow sinusoids generated by the production of mediators (such as the macrophage inflammatory protein-2 [MIP-2], granulocyte-colony stimulating factor [G-CSF], and c-x-c motive chemokine 1 [CXCL1]) (7, 10). The precise mechanism by which inflammation leads to circulating neutrophilia is not yet fully understood. Nevertheless, acute mobilization of neutrophils from the bone marrow may require the coordinated, yet unambiguous, actions of G-CSF and CXC chemokines, whereby G-CSF disrupts the retention mechanisms in the bone marrow (such as the CXCR4-stromal-derived-factor-1 axis) (7, 13). Besides the participation in the release of mature PMNs from the bone marrow, G-CSF is the principal regulator of physiological granulopoiesis. Effects include commitment of progenitor cells to the myeloid lineage, proliferation of granulocytic precursors, and reduction in transit time across the granulocytic compartment (7, 14, 15). Because of this proliferation and the reduction in transit time, G−CSF is nowadays also used therapeutically for the pretreatment of granulocyte donors (16).

After their release from the bone marrow, PMNs circulate in the blood stream under physiological conditions for fewer than 24 hours. The short life span and high production rate of PMNs require the equivalent elimination of PMNs from circulation to maintain homeostasis (6). Therefore, a certain part of circulating PMNs undergo constitutive apoptosis. Apoptotic PMNs are sorted out in the bone marrow, liver, and spleen and subsequently eliminated by efferocytosis (17, 18).

Besides PMNs circulating in the blood (circulating pool), there are two or maybe three reservoirs in which PMNs are resting and can be released on demand.

First, the bone marrow contains a reserve of less mature (band-shaped) and mature PMNs, which are retained in the reserve by life-sustaining cytokines of the bone marrow (bone marrow pool). The prevailing idea in the literature is that neutrophils receive complex anti-apoptotic signals in the bone marrow, which are less present or even absent in the bloodstream. These signals may include G−CSF and GM-CSF, although GM-CSF has a much smaller effect on mouse neutrophils than G-CSF (19–21). Additionally, mesenchymal stem cells (MSC) of the bone marrow are likely to protect neutrophils from apoptosis, maintaining their effector functions and preventing the disproportionate activation of the oxidative metabolism. Thereby, the key MSC-derived soluble factor IL-6 has been shown to be responsible for neutrophil protection from apoptosis (22). Moreover, SerpinB1 (an inhibitor of neutrophil serine proteases NE, CG, and PR-3) seems to be essential for maintaining a healthy PMN bone marrow pool by preserving anti-apoptotic signals (23).

Second, some PMNs are not intravascularly located in the main blood stream but adhere loosely to the endothelium of venous blood vessels (marginated pool). By recruiting this reserve, the number of neutrophils in the blood stream can be rapidly increased (5, 7). Such marginated pools can be found in the bone marrow, liver, spleen, and, as currently discussed, also in the lungs (7).

The size of individual marginated pools is considered to be the product of the mean intravascular transit time through the organ (i.e. the mean time it takes for neutrophils to transmigrate the capillary bed) and its blood flow (7). Peters et al. and Ussov et al. quantified the mean neutrophil intravascular transit time for the bone marrow (10 min), spleen (10 min), and liver (2 min) (24, 25). Although the size of the marginated pulmonary pool is predominantly determined by the mean pulmonary transit time (approximately 3–6 min), exact specification is challenging and controversial (7, 26–30). It has been estimated that 49% of the total blood granulocyte pool resides in the circulating pool, whereas the remaining 51% of the pool is attributed to PMNs of the marginated pool (7, 31).

Until recently, medical education books, such as Janeway’s Immunobiology, stated that PMNs are not present in healthy tissue in contrast to other phagocytizing cells (32). However, very recent studies (reviewed in (4, 7)) have demonstrated that PMNs are also physiologically present in the interstitium of the organs in which marginated pools are observed, namely in the bone marrow, liver, spleen, and lungs (4). As reported recently, PMNs may also be found, albeit to a smaller degree, in uninfected lymph nodes, the intestine, white adipose tissue, the skin, and in skeletal muscles (33, 34). Nevertheless, the question how and why PMNs are physiologically concentrated in these tissues remains unanswered. On the one hand, it is possible that these organs are further reservoirs that can rapidly supply PMNs in case of emergency. On the other hand, organ-resident PMNs may patrol through the above-mentioned organs, searching for damaged tissue and micro-organisms (4). To develop this idea further, PMNs detecting a pathogen would be able to rapidly and directly activate the adaptive immune system by physical contact and communication with other organ resident immune cells. In line with this theory, Puga et al. showed that splenic PMNs are able to directly activate B cells even under physiological conditions (30, 35). Moreover, the interaction of organ-resident pulmonary PMNs with B cells appears to play an important role in regulating the immune response (30, 36).

For this purpose, PMNs seem to be endowed with important non-immune regulatory functions when migrating through healthy tissues (34). As shown by Doerschuk et al. and Downey et al., one mechanism for restricting the presence of PMNs in pulmonary capillaries is the need for neutrophils to deform (27, 28).

Currently, the question of why PMNs are present in healthy tissue has not been fully clarified yet. So far, it is not known which tissues are actually involved, which dynamic processes take place, and—maybe more decisively—to what extent infiltrating PMNs contribute to healthy tissue homeostasis (4, 34).

Besides the controversially discussed physiological presence of PMNs in tissue, PMNs migrate very quickly, either as a consequence of infections or sterile tissue damage, from peripheral blood into peripheral tissues to fight a lesion there (37). This process is called extravasation (38). In an acute inflammatory reaction, granulopoiesis in the bone marrow increases, and a large number of PMNs accumulate very rapidly at the site of infection or the lesion. In the process, the life span of the circulating PMNs also becomes significantly extended (3).

Inflammatory reactions are modulated by inflammatory mediators, which are released by sensitive leukocytes (such as macrophages, dendritic cells, or mast cells) in the tissue when pathogens or disturbed tissue homeostasis are detected. Mediators may also be released by endothelial cells, by epithelial cells, by fibroblasts or by PMNs themselves upon activation (4, 37, 39, 40).

The most important mediators are chemokines, peptides, and eicosanoids. Chemokines are a large group of chemotactic cytokines, which are divided into four groups according to the arrangement of the two N-terminal cysteine residues designated CXC, CC, C, and CX3C, depending on the spacing of the conserved cytokines (“X” stands for an amino acid). CXC chemokines mainly target neutrophils and lymphocytes, whereas CC chemokines target a variety of cell types including macrophages, eosinophils, basophils, and dendritic cells (see **Table 1**) (84, 85).

**Table 1:** Important inflammatory mediators and associated cell types..

Mediator	Human	Origin of mediators	Receptor	Affected cell type	Literature source
Systematic					
		**Chemokines**			
**CXCL-1**	**GRO-α**	Monocytes,	**CXCR2**	Neutrophils,	(37, 38, 41)
**CXCL-2**	**GRO-β**	fibroblasts,		naive T-cells,	
**CXCL-3**	**GRO-γ**	endothelium		fibroblasts	
**CXCL-5**	**ENA-78**	Epithelial cells,	**CXCR2**	Neutrophils, monocytes,	(37, 41–44)
		eosinophils		microvascular or endothelial cells	
**CXCL-6**	**GCP-2**	Macrophages, epithelial cells, mesenchymal cells	**CXCR1/CXCR2**	Neutrophils, monocytes, microvascular or endothelial cells	(37, 41, 45)
**CXCL-7**	**NAP-2**	Platelets	**CXCR2**	Neutrophils,	(37, 38, 46)
		NK cells		mesenchymal stem cells	
**CXCL-8**	**IL-8**	Monocytes,	**CXCR1**	Neutrophils,	(37, 38, 41)
		macrophages,	**CXCR2**	naive T-cells,	
		fibroblasts,		monocytes	
		epithelial cells,			
		endothelial cells			
**CCL-2**	**MCP-1**	Monocytes,	**CCR2B**	Monocytes,	(37, 38, 41)
		macrophages,		NK and T-cells,	
		fibroblasts,		basophils,	
		keratinocytes		dendritic cells	
**CCL-3**	**MIP-1α**	Monocytes,	**CCR1**	Monocytes,	(37, 38, 41)
		T cells,	**CCR3**	NK and T-cells,	
		fibroblasts,	**CCR5**	basophils,	
		mast cells		dendritic cells	
**CCL-4**	**MIP-β**	Monocytes,	**CCR1**	Monocytes,	(37, 38, 41)
		macrophages,	**CCR3**	NK and T-cells,	
		neutrophils,	**CCR5**	dendritic cells	
		endothelium			
**CCL-5**	**RANTES**	T-cells,	**CCR1**	Monocytes,	(37, 38, 41)
		endothelium,	**CCR3**	NK and T-cells,	
		platelets	**CCR5**	basophils,	
				eosinophils,	
				dendritic cells	
**CCL-7**	**MCP-3**	Peripheral blood, mononuclear cells	**CCR1**	Neutrophils,	(37, 41, 47–49)
			**CCR2**	monocytes, dendritic cells, T-cells	
			**CCR3**		
**CXCL-12**	**SDF-1**	Bone-marrow-derived stromal cells, mesenchymal cells	**CXCR4**	Widely expressed	(37, 41, 50, 51)
**CX3CL1**	**FRAKTAL-KINE**	Monocytes, microglial cells, endothelium	**CX_3_CR1**	Macrophages, endothelial cells, smooth-muscle cells, T-cells	(38, 41)
		**Peptides/Cytokines**			
**C5a**	Liver cells	**C5aR1** (CD88)	Platelets, neutrophils, eosinophils, monocytes, macrophages, dendritic cells, mast cells, lymphocytes, cardiomyocytes, astrocytes, microglia, neural stem cells, oligodendrocytes, synoviocytes, articular chondrocytes, hepatic kupffer cells, stimulated hepatocytes, keratinocytes		(37, 52–57)
			Cells of renal glomerulum, mesangium,		
			endothelium, bronchial epithelium		
		**C5aR2** (C5L2, GPR77)	Neutrophils, macrophages, immature dendritic cells		(37, 53–55, 57–59)
			Specific T-cell subsets		
			Cells of bone marrow, adrenal gland, spinal cord, thyroid, liver, lungs, spleen, brain and heart		
			Adipocytes, skin fibroblasts		
**C3a**	Liver cells	**C3aR**	Neutrophils, T-cells Dendritic cells		(37, 57, 60)
			NK cells, mast cells,		
			monocytes/macrophages,		
			tubular epithelium, glomerular podocytes		
**Formylated peptides** (e.g. fMLP)	Invading pathogens,	**FPR1**	Neutrophils, monocytes, macrophages, (myo)fibroblasts,		(37, 61–67)
	dead and dying host cells (passive release of mitochondrial formylated peptides)		cells of bronchial or colonic epithelium		
**Pro-Gly-Pro** (PGP)	Liberated from ECM collagen *via* MMPs and PE	**CXCR2**	Neutrophils,		(37, 68–70)
			cells of bronchial epithelium		
**LL37**	Neutrophils,	**FPR2**	Neutrophils, eosinophils,		(37, 71–75)
	cells of skin, lungs, and gut epithelium, mast cells, lymphocytes, monocytes		T-cells, mast cells		
**MIF**	Monocyte/macrophages, dendritic cells, B−cells, neutrophils, eosinophils,	**CXCR2**	Neutrophils, monocytes, T-cells		(37, 76–78)
	Basophils mast cells				
		**Eicosanoids**			
**Leukotriene B4** (LTB4)	Derived from arachidonic acid released from phospholipids in cellular membranes	**BLT1**	Neutrophils, macrophages, eosinophils, T-cells, epithelial/endothelial cells, fibroblasts, smooth muscle cells		(37, 79, 80)
**Platelet activating factor** (PAF)	Derived from arachidonic acid released from phospholipids in cellular membranes	**PAFR**	Neutrophils, T-cells, platelets, macrophage-lineage cells (M0, Kupffer cells and microglia), thoracheal cells of epithelium, endothelium, myometrium		(37, 81–83)

Sensitive leukocytes are activated by certain surface structures found on pathogens (pathogen-associated molecular patterns, PAMPs) or endogenously released from cells through inflammasome activation or passively after cell damage (damage-associated molecular patterns, DAMPs) (86, 87). PAMPs and DAMPs can be recognized by sensitive leukocytes *via* certain receptors (pattern recognition receptors, PRR). PRR-mediated activation of sensitive leukocytes induce the release of proinflammatory mediators, such as IL-1β, IL-6, TNF−α, and other specific neutrophil-active chemo-attractants (see chapter 5) (37, 88).

These mediators trigger the recruitment of leukocytes to inflammatory tissues, regulate cell death in inflammatory tissues, induce the production of acute-phase proteins, and modify vascular endothelial permeability (86). In the specific case of PMNs, mobilization from the bone marrow into the blood stream is regulated by the mediators leukotriene B4, active complement component C5 (C5a), and the interleukin C-X-C motif chemokine-ligand 8 (CXCL8, formerly also called IL-8), which are released by the mechanisms described above. Furthermore, these mediators direct PMNs to the lesion site *via* chemotactic gradients (for details on chemotaxis, see chapter 5) and finally induce them to leave the blood and migrate into the surrounding tissue (extravasation) (38, 89).

## 2 The Process of Extravasation

**Figure 3:**
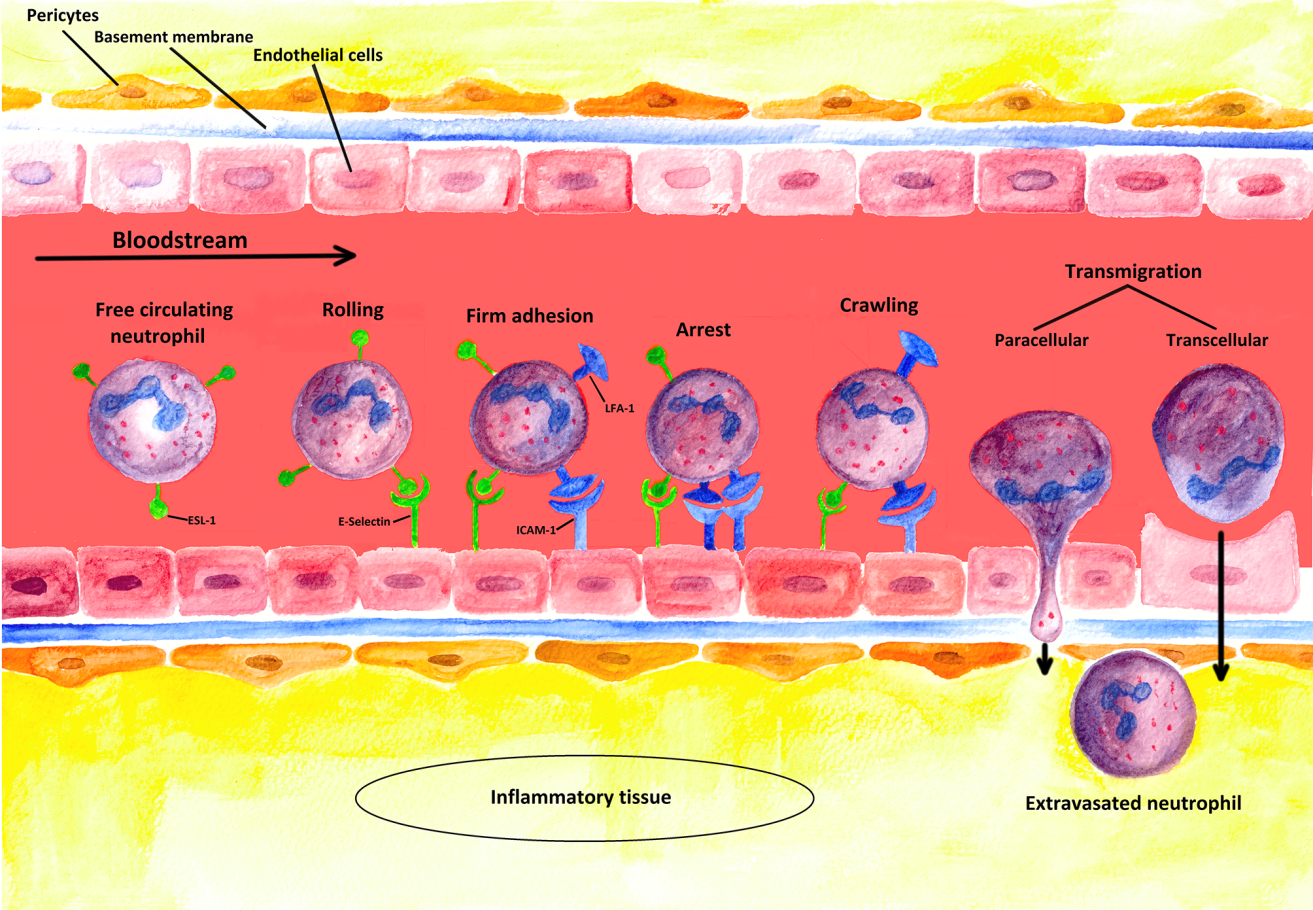
Schematic illustration of the extravasation process: PMNs leaving a blood vessel through the endothelium. The first step of the multi-stage process is the weak binding of PMNs to the endothelium due to interactions between selectins induced on endothelial cells and their corresponding ligands on the PMNs. In this figure, the process is illustrated for E-selectin and its ligand ESL-1 [containing sialyl-Lewis^x^-unit (s-Le^x^)]. However, such binding is not strong enough to resist the shear forces of the blood flow, so that new bondages are continuously formed and released again (rolling). Stronger interactions are only induced, however, when a chemokine (such as CXCL-8) binds to its specific receptor (not shown) on the neutrophil cell, which triggers the activation of the integrins LFA-1 and CR-3 (Mac-1) (firm adhesion). To induce the expression of adhesion molecules [such as ICAM-1 (ligand of LFA-1)] on the endothelium, inflammation-specific cytokines such as TNF-α are additionally required. Strong binding between ICAMs and integrins terminates rolling (arrest) and allows PMNs to squeeze between the endothelial cells (paracellular transmigration); yet, a transcellular way of transmigration is also possible as described in the literature. The neutrophil cell then crosses the basement membrane with the help of matrix metalloproteinases (like MMP-9), which are expressed on the neutrophil cell surface. Finally, the extravasated PMN migrates along a concentration gradient of chemokines secreted by cells at the sites of infection in the interstitium (4, 32).

The recruitment of PMN requires adhesion to and subsequent transmigration through vascular walls (see **Figure 3**) (90). In most tissues, PMNs leave the vascular system through postcapillary venules (91). Only in the lungs does the extravasation process occur through capillaries (91).

The initial action of PMN extravasation is the activation and upregulation of adhesion molecules in the endothelium situated in close proximity to inflammatory tissue (37). As described, such activation can be mediator-induced. However, the endothelium itself can also recognize PAMPs and DAMPs *via* its own PRRs (4). The decisive point is that adhesion molecules are upregulated in both ways, mediator-induced and endothelium-induced. This process is crucial for initiating the recruitment of neutrophils (89).

The most important adhesion molecules for the recruitment process are P- and E-selectins. P−selectins are physiologically stored in the Weibel-Palade bodies in dormant endothelial cells and in α-Granula in platelets. When activated, P−selectins can be immediately relocated to the apical cell membrane. E-selectins, however, are *de novo* synthesized, and appear on the endothelial surface within 90 min (4, 92). Having reached the endothelial surface, the selectins bind to the adhesive ligands present on the PMNs (89). Both selectins bind to the sialyl-Lewis^X^ unit, an oligosaccharide present on the cell surface protein of circulating PMNs (38). E−selectin preferentially binds to E-selectin ligand-1 (ESL-1), whereas P-selectin mainly binds to P-selectin glycoprotein ligand-1 (PSGL−1, CD162). Both ligands have sialyl-Lewis^X^ units (93).

Owing to the Fåhraeus-Lindqvist effect, cellular components are usually located in the center of small blood vessels, in which flow velocity is at its maximum. In inflammation foci, blood vessels are dilated. The resulting lower flow velocity enables PMNs to interact more easily with the endothelial surface by the mechanism just described. As a consequence, PMNs that are freely circulating in the blood stream become attached to the endothelial surface. This first interaction of P- and E-selectins with their ligands (see above), however, cannot anchor the cells against the shear forces of the blood stream. Subsequently, the cells “roll” by reversible binding along the endothelium by constantly making and breaking contact with the endothelium and the cells (for details see chapter 3) (38, 89, 94, 95).

As a next step, G-protein-coupled receptors on the “rolling” granulocytes bind to PMN attractants secreted on the apical membrane (for details, see chapter 3 and 5) (89). By this binding, an “inside-out signal” is transmitted to the PMNs, which causes conformational changes in the PMN surface proteins termed β2-integrins. Of particular importance here are the two continuously expressed β2-integrins lymphocyte function-associated antigen 1 (LFA-1) and macrophage antigen 1 (MAC-1; alternative name: complement receptor 3, CR3) (4).

LFA-1 und MAC-1 interact with the intercellular adhesion molecules ICAM-1 and ICAM−2 on the endothelial surface (4). Usually, LFA-1 und MAC−1 bind their ligands only weakly (38). Due to the conformational change, however, LFA-1 und MAC-1 bind very firmly to the ICAMs, causing the end of the “rolling” and firm adhesion of the PMNs to the endothelium (“arrest”) (38).

Besides G-protein signaling, the conformational activation of LFA-1 required for neutrophil arrest can be induced by selectin engagement (96). On the one hand, E-selectin binding to its ligands on PMNs supports slow rolling and facilitates activation of high-affinity β2-integrins. Thereby, bond formation with ICAMs leads to PMN arrest on inflamed endothelium (97–101). On the other hand, a study published by Morikis et al. in 2017 showed the important role of L−selectin in transitioning neutrophils from rolling to arrest, which led to a paradigm shift in understanding mechanosignaling of human PMNs during recruitment (97, 102). E-selectin ligation on L-selectin and PSGL-1 receptors induces their redistribution into membrane clusters (97, 98). The PSGL-1/L-selectin complex signals through Src family kinases, ITAM domain–containing adaptor proteins, and other kinases, which ultimately results in LFA-1 activation (96).

It is noteworthy that, in neutrophil arrest, G-protein-coupled and selectin-mediated outside-in signaling can effectively amplify the number of high-affinity β2-integrins (103). Thus, cooperation of both signaling ways is temporally required for regulating the number and affinity state of β2-integrins (97).

After neutrophil “arrest”, PMNs actively “crawl” to suitable endothelial passageways (“crawling”) (4). Such “crawling” is based on the firm binding of MAC−1 to ICAM-1 (104). These bindings maintain adhesion to the endothelial surface at all times, thus enabling the PMNs to “crawl” perpendicularly along the endothelium or against the blood flow under the shear conditions of the blood stream until they reach the preferential site of transmigration or a passageway (4, 104, 105).

To finally leave the blood vessels, PMNs must first pass through the endothelium (transmigration). Suitable passageways are located at the cell-to-cell junctions between endothelial cells. Of particular importance during paracellular transmigration through the endothelium is the binding of the integrins LFA-1 und MAC-1 to the cellular adhesion molecules ICAM−1 and ICAM−2 or to the vascular cell adhesion protein 1 (VCAM−1). However, other adhesive interactions involving junctional proteins such as the platelet endothelial cell adhesion molecule-1 (PECAM1; alternative name CD31) are also important. All these interactions finally allow PMNs to force their way through the endothelium (4, 38).

However, neutrophil recruitment does not follow this classical cascade in every organ and may sometimes require organ-specific mechanisms (4, 106, 107). For instance, neutrophils recruited into an inflamed liver appear to lack rolling and to adhere directly due to the interaction of CD44 on PMNs and hyaluronan (HA) on liver sinusoidal endothelium (106–109). In the lungs, PMNs mainly exit vessels at the alveolar capillary level; thus, PMN rolling is unlikely and seems to be replaced by mechanical sequestration because cytokine-induced cytoskeleton-dependent PMN stiffening due to F-actin polymerization provokes dramatic slowing of PMNs within narrow-caliber capillaries (110). Moreover, neutrophil recruitment in the brain seems to depend on the presence of platelets adhering to the endothelium and building a “bridge” between the endothelium and the PMNs (4, 111). **Table 2** shows receptors and corresponding ligands that are important for the neutrophil extravasation process.

**Table 2:** Important receptors and corresponding ligands involving neutrophil adhesion and signaling..

Tissue	Receptor Family	Receptor	Cell Type of Receptor	Ligand on Neutrophils		Literature Source
**Tethering and rolling**						
**Postcapillary venules**	Selectins	**P-selectin** (PADGEM, CD62P)	Activated endothelium, platelets	**PSGL-1** (CD162) (positively regulates recruitment)		(4, 38, 112)
				**PTX-3** (negatively regulates recruitment)		
		**E-selectin** (ELAM-1, CD62E)	Activated endothelium	**PSGL-1** (CD162)**, ESL−1** (GLG-1)**, CD44**		(4, 38, 112)
		**PSGL-1** (CD162)**, GlyCAM**	Activated endothelium	**L-selectin** (CD62L)		(4, 38)
**Liver sinusoids**	**This step does not occur **					
**Brain venules**	Selectins	Platelet-dependent:	Activated endothelium, platelets	**PSGL-1** (CD162)		(4, 38, 112–116)
		**P-selectin** (PADGEM, CD62P) or				
		**E-selectin** (ELAM-1, CD62E)				
	Immunoglobulin superfamily	**VCAM-1** (CD106)	Activated endothelium	P-Selectin dependent: **VLA-4** (α_4_β_1_-Integrin)		(4, 38, 117–119)
**Lung venules**	Selectins	**P-selectin** (PADGEM, CD62P)	Platelets	**PSGL-1** (CD162)		(4, 38, 110, 120)
		**E-selectin** (ELAM-1, CD62E)	Activated endothelium,	**PSGL-1** (CD162)		(4, 38, 110, 120)
			platelets			
		?		**L-selectin**		(4, 38, 110, 121)
				(CD62L)		
**Slow rolling**						
**Postcapillary venules**	Immunoglobulin superfamily	**ICAM-1** (CD54)	Activated endothelium, activated leukocytes	PSGL-1-induced: **LFA-1** (α_L_β_2_-Integrin, CD11a/CD18)		(4, 38)
	Selectins	**E-selectin** (ELAM-1, CD62E)	Activated endothelium	**PSGL-1** (CD162)**, ESL−1** (GLG-1)**, CD44**		(4, 38, 122)
**Liver sinusoids**	**This step does not occur**					
**Brain venules**		?		?		(4, 122)
**Lung venules**	Immunoglobulin superfamily	**ICAM-1** (CD54)	Activated endothelium, activated leukocytes	PSGL1-induced: **LFA-1** (α_L_β_2_-Integrin, CD11a/CD18)		(4, 110, 123)
**Arrest and Adhesion**						
**Postcapillary venules**	Immunoglobulin superfamily	**ICAM-1** (CD54)	Activated endothelium, activated leukocytes		**LFA-1** (α_L_β_2_-Integrin, CD11a/CD18)	(4, 38)
		**VCAM-1** (CD106)	Activated endothelium		**VLA-4**	(4, 38)
**Liver sinusoids**	Immunoglobulin superfamily	**ICAM-1** (CD54)	Activated endothelium, activated leukocytes		**MAC-1** (α_M_β_2_-Integrin, CR3, CD11b/CD18)	(4, 38)
	Glucosamino-glycan	**Hyaluronan**	Activated endothelium		**CD44**	(4, 106, 108, 124)
	Enzyme (Peptidase)	**DPEP-1**	Activated endothelium		**LSALT**	(106)
**Brain venules**	Immunoglobulin superfamily	**ICAM-1** (CD54)	Activated endothelium, activated leukocytes		**LFA-1** (α_L_β_2_-Integrin, CD11a/CD18)	(4, 38, 125)
**Lung venules**	Immunoglobulin superfamily	**ICAM-1** (CD54)	Activated endothelium, activated leukocytes		**MAC-1** (α_M_β_2_-Integrin, CR3, CD11b/CD18)	(4, 38, 110, 126, 127)
		**VCAM-1** (CD106)	Activated endothelium		**VLA-4**	(4, 38, 110, 128)
	Enzyme (Peptidase)	**DPEP-1**	Activated endothelium		**LSALT**	(106)
**Crawling**						
**Postcapillary venules**	Immunoglobulin superfamily	**ICAM-1** (CD54)	Activated endothelium, activated leukocytes		**MAC-1** (α_M_β_2_-Integrin, CR3, CD11b/CD18)	(4, 38)
**Liver sinusoids**	Immunoglobulin superfamily	**ICAM-1** (CD54)	Activated endothelium, activated leukocytes		**LFA-1** (α_L_β_2_-Integrin,	(4, 38, 129)
					CD11a/CD18);	
					**MAC-1** (α_M_β_2_-Integrin, CR3, CD11b/CD18)	
**Brain venules**	Immunoglobulin superfamily	**ICAM-1** (CD54) **ICAM-2** (CD102)	Activated/resting endothelium, activated leukocytes, dendritic cells (ICAM-2)		**LFA-1** (α_L_β_2_-Integrin,	(125, 129)
					CD11a/CD18);	
					**MAC-1** (α_M_β_2_-Integrin, CR3, CD11b/CD18)	
**Lung venules**	Immunoglobulin superfamily	**ICAM-1** (CD54)	Activated endothelium, activated leukocytes		**MAC-1** (α_M_β_2_-Integrin, CR3, CD11b/CD18)	(4, 110, 130)
**Transmigration and Diapedesis**						
**Postcapillary venules**	Immunoglobulin superfamily	**VCAM-1** (CD106)	Activated endothelium		**VLA-4**	(4, 38)
		**ICAM-1** (CD54)	Activated/resting endothelium, activated leukocytes		**LFA-1** (α_L_β_2_-Integrin,	(4, 38)
					CD11a/CD18);	
		**ICAM-2** (CD102)			**MAC-1** (α_M_β_2_-Integrin, CR3, CD11b/CD18)	
		**PECAM-1** (CD31)	Activated leukocytes, endothelial cell-cell junctions		**PECAM-1** (CD31)	(4, 38)
		**JAM-A**	Activated endothelium		**LFA-1** (α_L_β_2_-Integrin, CD11a/CD18)	(4, 38, 110, 131)
		**JAM-B**	Junctions at interendothelial contacts		**VLA-4** (α4β1-Integrin)	(4, 132, 133)
		**JAM-C**	Junctions at interendothelial contacts		**MAC1** (α_M_β_2_-Integrin, CR3, CD11b/CD18)	(4, 134, 135)
	Membrane glycoprotein	**CD99**	Activated endothelium, activated leukocytes		**CD99**	(4, 136, 137)
		**CD99L2**	Activated leukocytes, cell contact between endothelial cells		**CD99L2**	(4, 136, 137)
	Calcium-dependent transmembrane glycoprotein	**VE-cadherin** (ESAM): negatively regulates recruitment	Activated/resting Endothelium		Between endothelial cells	(4, 138–140)
	Glucosamino-glycan	**Hyaluronan**	Activated/resting endothelium		**CD44**	(4, 124)
**Liver sinusoids**	Immunoglobulin superfamily	**ICAM-1** (CD54)	Activated endothelium, activated leukocytes		**MAC-1** (α_M_β_2_-Integrin, CR3, CD11b/CD18)	(4, 38, 141, 142)
**Brain venules**	Immunoglobulin superfamily	**PECAM-1** (CD31)	Activated leukocytes, endothelial cell-cell junctions		**PECAM-1** (CD31)	(4, 38, 125, 143)
**Lung venules**	Calcium-dependent transmembrane glycoprotein	**VE-cadherin** (ESAM): negatively regulates recruitment	Activated/resting Endothelium		Between endothelial cells	(110, 138–140)
	Immunoglobulin superfamily	**JAM-A**	Activated endothelium		**LFA-1** (α_L_β_2_-Integrin, CD11a/CD18)	(110, 131, 144)
		**JAM-C**	Junctions at interendothelial contacts		**MAC-1** (α_M_β_2_-Integrin, CR3, CD11b/CD18)	(110, 134, 135)

Comment on **Table 2**: The classical cascade refers to neutrophils extravasating in postcapillary venules; in some organs, however, extravasation can take place in different vessels. In the liver, brain, and lungs, some steps of the classical cascade do not occur or require divergent adhesion molecules. “?” indicates unknown data (4, 145).

However, PMNs can also get past the endothelial layer on the transcellular pathway through endothelial cells. To what extent the endothelium actively participates in this transmigration has not yet been fully elucidated. The current assumption is that—at this point in the transendothelial migration process—, the endothelium actively participates by inducing actin-rich structures that surround transmigrating leukocytes, which extend dorsally in some cases (146, 147).

During extravasation, PMNs penetrate the vascular endothelial lining, which requires opening of the endothelial barrier. Remarkably, this process does not necessarily cause any plasma leakage. The questions how endothelial cells form transmigration areas through which PMNs can migrate and what mechanisms are behind the ability of the endothelium to prevent leakage and maintain integrity while numerous leukocytes are penetrating are still under investigation. Platelets docking to von Willebrand factor seem to be essential for closing endothelial gaps induced by transmigrating neutrophils through stimulating the angiopoietin receptor Tie-2 (148–150). Nevertheless, paracellular and transcellular pathways do coexist, but current data are contradictory in terms of which pathway is preferred by PMNs (151).

When PMNs reach the end of the endothelial cell layer, they must overcome the endothelial basement membrane, a passage termed diapedesis (38). The basement membrane is a continuous structure consisting of proteins of the extracellular matrix (ECM proteins) such as collagen (mainly collagen IV) and laminin. Neutrophils possess specific proteases with enzymatic activity against ECM proteins, which include matrix metalloproteases such as MMP-9 and serine proteases such as neutrophil elastase. Although one may easily conclude that PMNs “cut” their way through the basement membrane, this process has not been conclusively proven yet. Even if histological examination did not show any rupture of the basement membrane in inflammatory tissue, it is nevertheless currently assumed that PMNs preferentially migrate through areas of the basement membrane that have a low content of ECM molecules (<60% as compared to otherwise dense areas). Thereby, MMPs seem to provide assistance in the process (4).

After overcoming the basement membrane, PMNs subsequently migrate through the pericytic region before reaching the interstitium. Pericytes are cells that wrap around endothelial cells, thus forming an interface between the circulating blood and the interstitial space. Interestingly, gaps in pericytic regions overlap with regions with lower basement membrane density. In the extravasation process, PMNs are therefore assumed to choose the path of least resistance when migrating to the interstitium (4).

## 3 Neutrophil Endothelial Adhesion and Rolling Mechanisms

**Figure 4:**
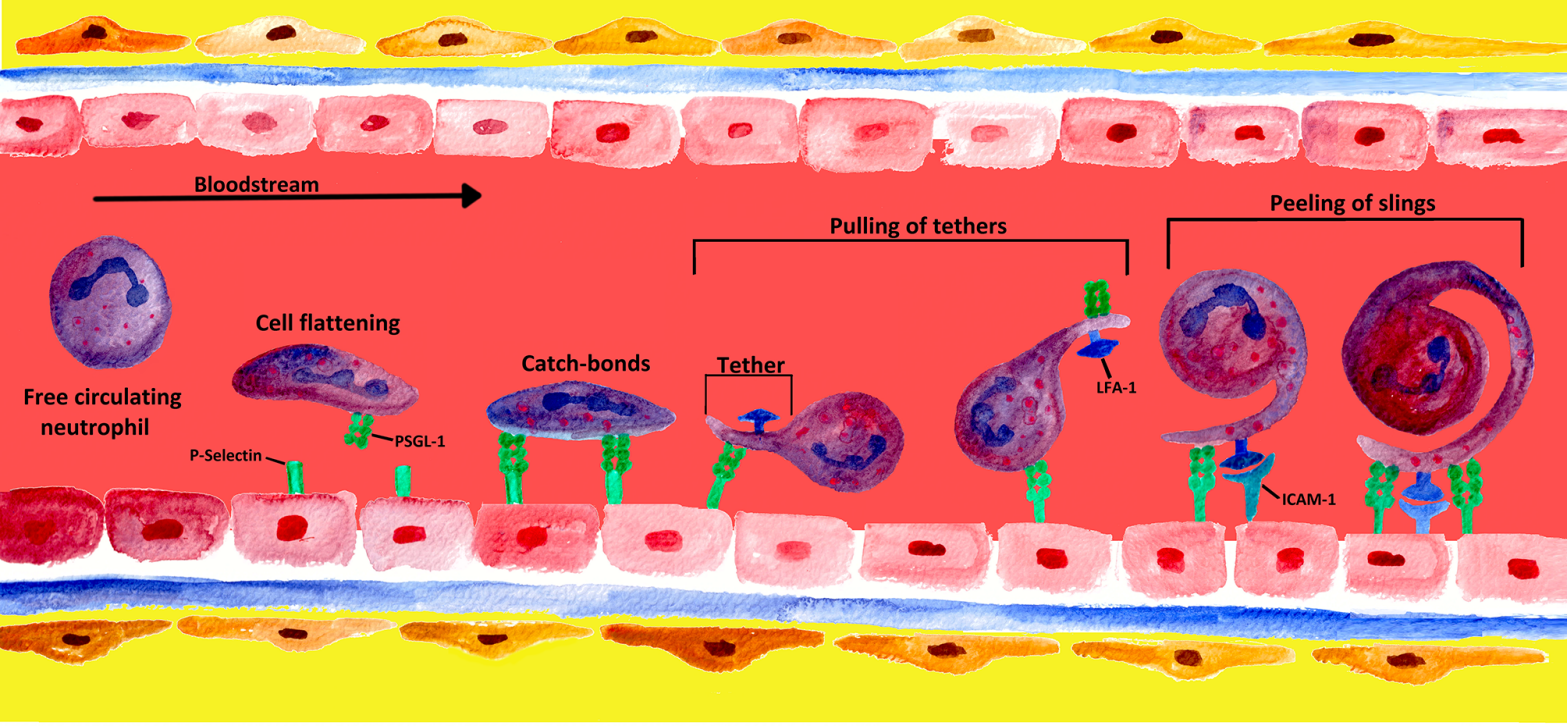
Mechanisms of formation and engagement of tethers and slings by rolling neutrophils. Rolling neutrophils experience high shear stress in the blood stream and have to overcome tensile stretch due to rolling. When PMNs converge into a blood vessel wall, the shear stress of the blood leads to cell flattening. PMNs limit stress forces during the rolling process by adhesive bonds generated at the front and disrupted at the rear of the PMN (4). At low detachment forces, these adhesive P-selectin-PSGL-1 bonds behave like catch bonds. With increasing force, the bonds become stronger, and long membrane bonds called tethers are created at the rear of the PMN (153, 154). The tethers bind to endothelial P−selectin *via* PSGL-1, forming temporary anchorage points that are subsequently disconnected from the endothelium by the pulling of tethers (4, 155). Once the tethers break at the rear of the rolling PMN, they swing forward and wrap around the cell as a sling, thereby decelerating the PMN. On slings, multiple patches along the whole projection are formed *via* the binding of PSGL-1 to the endothelium. This sequential attachment and pulling apart is referred to as the “step-wise peeling of slings”. The final deceleration and arrest of the cell results from the interaction of neutrophil LFA-1 with endothelial ICAM-2, leading to an even tighter wrapping of the sling around the cell body (4, 123).

In contrast to most leucocytes, which—for the most part—are only able to roll along the walls of venules at low shear stress, neutrophils have the ability to roll at a 10-fold higher shear stress level (152). Although the mechanisms are not yet completely understood, four potential mechanisms have been identified that enable neutrophils to roll at high shear stress of the bloodstream: cell flattening, catch bond behavior, membrane tethers, and slings (see **Figure 4**) (153).

At high shear stress in post-capillary venules, rolling neutrophils deform into a tear drop shape and undergo flattening. This process is the result of elongation in the flow direction imposed by the hydrodynamic drag acting on the rolling cell. On the one hand, this process decreases cell height and subsequently reduces the hydrodynamic drag experienced by the rolling cell. On the other hand, as the cell flattens, the contact area or cell footprint on the vessel wall is increased, raising the probability of P-selectin-PSGL-1 bond formation (153, 156–159).

Neutrophils rolling along the vascular endothelium are mainly a result of rapid formation and dissociation of P-selectin-PSGL-1 bonds at the center and rear of the rolling cell, which balance the hydrodynamic drag of the blood stream (152). P-selectin-PSGL-1 bonds behave like catch bonds at small detachment forces and thus become stronger with increasing force (153, 154).

Nevertheless, PSGL-1 does not only bind to P-selectin but is also one of the major ligands of L-selectin (CD62L) (160, 161). Unlike E- and P-selectin, which are expressed on activated endothelium (see chapter 2), L-selectin is the only selectin constitutively expressed at the tips of microvilli on PMNs (162, 163). L-selectin undergoes split second changes in bond lifetime with its ligand (most likely PSGL-1 with sialyl-LewisX unit) under flow conditions, classified into catch and slip bonds (102). Interestingly, L-selectin on human neutrophils is loaded itself with sialyl-LewisX, whereby it can be recognized by E-selectin (164). Therefore, L−selectin is one of the first neutrophil adhesion molecules to be in contact with the endothelium under flow conditions (165).

During early adhesion, initial contact between the calcium dependent (C-type) lectin domain (CTLD) of neutrophil L-selectin and its ligand exerts low tenacity, which starts at the leading edge of the cell (slip bond). At low shear stress (<0.3 dyn/cm^2^), slip bonds usually last less than a second, and their lifetimes are shortened by force (102, 154, 166). As shear stress rises up to an optimum level (∼1.0 dyn/cm^2^), the tenacity between the CTLD and its ligand increases to unfold. Increasing force prolongs bond lifetimes (catch bonds), now located at the trailing end of the cell. Above the optimum level of shear stress, force shortens bond lifetimes (slip bonds); when the tenacity exceeds the limit for catch bonds, bond lifetime decreases, and CTLD and its ligand separate again (102, 154, 166, 167). Under conditions of abundant ligand availability, a new catch bond will form at the new leading edge to repeat the process, culminating in classic cell rolling behavior (102).

In contrast to E- and P-selectin, L-selectin is rapidly cleaved from the cell surface in response to cellular activation, inflammatory stimuli, and mechanical force, a process termed “shedding” (162, 168, 169). Although many issues regarding the physiologic role of shedding remain unanswered, this process seems to play a key role in regulating neutrophil rolling and adhesion dynamics mediated by L-selectin-ligand interactions (168–171).

In summary, increasing force leads to triphasic (slip-catch-slip) behavior of selectin-ligand interactions and lifetimes (154, 166, 167, 172–174). *In vitro* and *in silico* studies have shown that such catch-behaving bonds stabilize neutrophil rolling at low shear stresses of less than 1 dyn/cm^2^ (160, 175–178). Nevertheless, no study has yet analyzed the role of catch-behaving bonds in facilitating neutrophil rolling at shear stresses higher than 6 dyn/cm^2^ (153).

Moreover, neutrophils rolling at high shear stresses form membrane tethers, which can be longer than the cell diameter and promote the survival of P-selectin-PSGL-1 bonds (153). Membrane tethers are nano-tubes extruded from the lipid bilayer membranes of blood cells. These tethers are formed when a microvillus on the surface of a neutrophil is pulled with force that is increased over time (179). Sundd et al. showed that such long membrane tethers, attached to the substrate *via* highly strained P-selectin-PSGL-1 bonds, contribute to the catch-bond behavior of the system (159, 180). Such bonds tend to increase their lifetime in response to the pulling force, thus allowing the tethers to stay attached to the P-selectin substrate for a longer time and to grow in length (153).

As mentioned above, blood flow imposes a hydrodynamic drag on the rolling cell, enabling the cell to move forward and to also rotate like a ball along the vessel wall. To continue rolling, the cell needs to at least partially balance both the forward and the rotating components of the hydrodynamic drag (180). Membrane tethers in cooperation with the catch bond phenomenon extend under pulling force and appear as “slings” at the front of the rolling cells (153, 180).

According to Sundd et al., neutrophil rolling at shear stresses of 6–10 dyn/cm^2^ is facilitated by slings, which are cell-autonomous adhesive structures extended at the front of rolling neutrophils. As the cell rolls over the sling laid in front of it, the sling starts to wrap around the rolling cell, which undergoes a step-wise peeling process at the rear of the cell due to the tandem failure of PSGL-1 patches under the hydrodynamic drag (180). When a PSGL-1 patch on a peeling sling fails, the cell tries to jump forward, but only for a short distance until the next patch downstream of the first patch on the same sling becomes loadbearing (180). This step-wise peeling distinguishes a tether from a sling because unlike the failure of a sling, failure of a tether is catastrophic as there are no other bonds available that can keep the tether attached to the substrate; thus, the cell accelerates forward (153).

The patchy distribution of PSGL-1 along each sling provides a unique adhesive substrate once the cell rolls over the sling. As each PSGL-1 patch fails, a new patch is already lined up that now becomes loadbearing. This step-wise peeling makes slings even more effective than tethers in slowing down rolling neutrophils (180).

Unlike PSGL-1, LFA-1 is expressed all over the neutrophil surface and the entire length of the slings (180). Although, LFA-1-ICAM-1 bonds have been shown to behave as catch-like bonds at small bonding forces, there is no report of such behavior of LFA-1-ICAM-2 bonds (181). Besides stabilizing, rolling slings are unique structures that also enable rolling neutrophils to present LFA-1 to their ligand ICAM-2. However, catch-like LFA-1-ICAM-2 interactions will probably result in even tighter wrapping of slings at smaller bonding forces compared to slip bonds. Eventually, the long tethers detach from the substrate and transform into slings, which stabilize rolling by undergoing a step-wise peeling process (180). As rolling progresses, the number of slings increases, which may explain the well-known phenomenon of rolling to become more stable over time (180, 182).

Interestingly, circulating PMNs can not only tether and role on the endothelium but also on adherent leukocytes. This process is termed “secondary tethering and rolling” and is enabled by interaction of the sialyl-LewisX unit of PSGL-1 with L-selectin. Secondary tethering extends PMN recruitment when endothelial cell-derived ligands are already masked by adherent leukocytes (151, 165, 173, 183). By promoting primary tethering and rolling, PSGL−1/L-selectin may contribute to chronic inflammation (165, 184–186).

Taken together, catch bonds, long tethers, cell flattening, and slings act together and contribute to the forces balancing the hydrodynamic drag, which may explain why neutrophils can roll even at very high shear stress as observed in acute inflammation *in vivo* (153, 180). How the synergy between the four mechanisms leads to stable rolling and whether catch-behaving bonds are responsible for formation of long tethers and slings are topics that need to be investigated further (153).

## 4 Migration in the Interstitium of the Target Tissue

After completing transendothelial migration, PMNs reach their target tissue. Once arrived at their target, PMNs migrate through the inflammatory interstitium along a chemokine gradient (see chapter 5) to reach their final destination (187). Depending on the site of the damage, PMNs can encounter very different tissues, such as fibrillar networks, cell-rich environments of an organ parenchyma, or lymphatic tissues (188). To be able to migrate through different tissues, PMNs preferentially use an amoeboid mode of locomotion, which is characterized by smooth and fast migration (189).

Crucial for this mode of locomotion is active cell body deformation. In PMNs, the intracellular forces of such deformation are almost exclusively generated by the actin-myosin cytoskeleton and characterized by the alternation of an intracellular network extension by actin polymerization followed by network contraction through actin-myosin. On the one hand, this contractility generates hydrostatic pressure on the rear side, which compresses cytoplasmic material and pushes it forward. On the other hand, adhesions at the rear edge of the cells are released (188). To move the cell, the cytoskeletal forces must be transferred to the ECM. The transfer can be integrin-mediated by the weak interaction of adhesion molecules, whereby, in contrast to the dominant participation of β2-integrins in the extravasation process, the interstitial migration process seems to be mainly associated with the activation of β1−integrins (91, 190, 191).

In addition, Nourshargh et al. described that, when integrin receptors are missing or unable to bind to the substrate, PMNs could also physically interact with the extracellular environment and thus achieve force transmission. The authors postulated that the possibility to use both modes of locomotion, i.e. integrin-dependent and integrin-independent locomotion, enables PMNs to migrate through a wide variety of different interstitial tissues (188).

Accordingly, Wolf et al. considered every single migration step in the interstitium as adaptive in response to cell-intrinsic signals and extracellular chemical and mechanical signals (regulation of adhesion, cytoskeletal dynamics, proteolysis, forming of the cell body, or geometry of the ECM) (192).

Friedl et al. summarized cell migration within the interstitial tissue as a complex mechano-chemical process that requires the interaction of key processes of the signaling, cytoskeletal, membrane, and adhesive systems (193).

## 5 Current Knowledge and Controversies of the Influence of the Extra-Cellular Matrix on the Function of Neutrophils

The mode of locomotion in the interstitium significantly differs from the mode of locomotion during extravasation. In the latter, cells remain firmly integrated into a tissue context by cell-to-cell or cell-to-ECM adhesion (188). The amoeboid mode of locomotion in the interstitium, however, is characterized by the absence of such strong adhesive interactions (194). Although intravascular events and transmigration through the endothelium have been comprehensively examined in numerous studies, comparatively relatively little interest has been paid to the steps after the extravasation cascade. As a consequence, the mechanisms regulating the passage through the interstitium are less well characterized (190). Cell-matrix interaction is not completely clarified (188), and little is known about the adhesive interactions determining the motility of migrating leukocytes in the interstitium (190).

The question whether and to what extent PMNs use extracellular conditions as guidance structures is not finally answered (188). Furthermore, the literature contains different and sometimes contradictory information as to whether the composition of the ECM influences the functions of granulocytes: Nourshargh et al. reported in their review that the amoeboid locomotion of leukocytes and thus also that of PMNs is independent of the composition of the extracellular environment (188). In contrast, in reference to a study by van Goethem et al. in which the migration of macrophages was influenced by ECM conditions, Jennings et al. postulated that PMNs adapt their mode of locomotion to the composition of the ECM. Jennings et al. assumed that the ECM environment encountered by PMNs determines the input of β1- or β2-integrins as well as the actin polymerization and the myosin-II-driven forces of the locomotion behavior (195).

Burns et al. gave an overview of how the adhesive properties of different ECM elements influence both the direction and speed of leukocyte movement. In addition, the authors described integrin-mediated adherence to the ECM as being very important for PMN locomotion towards inflammatory sites (91).

Relying on previous studies by Cox and Huttenlocher (196), Lindbom et al. endorsed the concept that repeated cycles of temporary adhesion to and detachment from matrix structures are necessary for the effective motility of PMNs. Integrins seem to be crucial here in so far that they establish contact with matrix molecules, thus enabling locomotion by acting as an anchor for the filaments of the cytoskeleton. Of course, there is also integrin-independent PMN migration (196), but such adhesion-independent mechanisms are far from being able to achieve significant effectiveness of the locomotor system under physiological conditions (190, 196). Furthermore, Lindbom et al. stated that the chemotaxis of granulocytes is also influenced by the relative frequency of matrix proteins within the tissue. Conditions in the extracellular environment can increase the binding strength between integrins and their ligands, thus antagonizing motility (190). Kuntz et al. assumed that migration must depend on adhesion to the ECM (197).

The previous findings on the influence of the ECM on PMN migration can be summarized insofar that the migration patterns determined by the ECM seem to be more modulated rather than strictly determined (188). The idea that the ECM can have a structural function by serving as a barrier or scaffold for cells infiltrating inflamed tissue is per se easy to imagine. Besides the influence on the migration of immune cells, Sorokin et al. also reported an influence of the ECM on the inflammation of tissues. As mentioned above, by binding chemokines in a spatially structured and regulated manner, the ECM can integrate and deliver multiple complex signals to leukocytes, which influences their behavior in inflammatory tissues (198). In line with this fact, we observed in a recent study that the ECM does not only impact neutrophil migration but also neutrophil immunological functions, such as ROS production, MPO release, and NETosis (199). Moreover, chemokines in inflammatory tissues increase both the tissue turnover and protease secretion of tissue-resident cells (198). Several publications—*inter alia* by Houghton et al. and by Ospelt and Gay—suggested that such aberrantly expressed ECM molecules can influence the activation, differentiation, and survival of immune cells (200, 201). Gaggar et al. described that the release of MMP8 and MMP9 by PMNs during an inflammation breaks down collagen into bioactive ECM fragments, which in turn have chemotactic activity (202). Weathington et al. provided evidence of such chemotactic activity in an *in vivo* lung inflammation model, thus proving the physiological relevance of ECM influence on inflammation and in particular on the function of PMNs (203). Nissen et al. demonstrated that, in the presence of bioactive fragments of collagen, PMNs produce less ROS and reduce their interstitial velocity of migration *in vitro*. Above all, Nissen et al. asserted in an *in vivo* asthma model that the same bioactive fragments selectively inhibit the accumulation of PMNs in lung interstitium, thereby proving the (patho−)physiological relevance of ECM influence on inflammation and especially on PMN functionality (204).

In the last 5 years, increased interest in the interaction of neutrophils with their surrounding ECM has advanced *in vivo* research in this matter. Both the presence of ECM and the interplay between neutrophils and their ECM are now considered as a vital mechanistic aspect of inflammation. There is mounting evidence of complex interactions between ECM macromolecules and PMN (reviewed recently by Zhu et al.). We now know that the close relationship between ECM and PMNs plays an important role in the progression of various diseases in humans (205). Recent studies for example demonstrated an important role for ECM in fighting against infectious diseases by mediating an antifungal response of PMNs (205–207). Moreover, experimental studies have shown that released NETs cleave fibronectin *via* NE and MMP-9 to further degrade ECM in alveoli, thereby promoting the development of bronchopulmonary dysplasia (205, 208). In addition, the interactions of PMN with ECM play a fundamental role in inflammatory conditions of many organs like myocardial injury and pulmonary diseases (205, 209, 210). Furthermore, there is a growing evidence that neutrophil invasion into tumor ECM is associated with cancer progression and subsequent metastatic dissemination (for details see *12 Neutrophil Behavior in Cancer Environment and Tumor Tissue*) (205, 211–214). Nevertheless, ECM-neutrophil interactions do have the potential for treatment options of PMN-associated diseases. However, gaps remain in understanding the regulatory role of ECM in determining neutrophil function. Hence, future studies are required to fill the gaps and decover underlying mechanisms, which could be used to treat patients with PMN associated diseases (205).

## 6 Chemotactic Signal Transduction of Migration

To be able to perform their functions adequately, PMNs must know the exact location of the lesion focus. Therefore, the targeted guidance of PMNs from the reservoirs through the vascular system to the affected tissue is of crucial importance (89). For this effective response, PMNs can detect extracellular chemotactic concentration gradients and move up the gradients towards higher concentrations. This process is referred to as chemotaxis (215, 216).

Neutrophil chemotaxis is characterized by three different processes: gradient detection, polarization, and cell motility (217). PMNs have receptors for chemokines and chemo-attractants, such as the endogenous molecules C5a, LTB4, and CXCL8 released in the course of an inflammatory response, but also for exogenous molecules such as the peptide N−formylmethionine-leucyl-phenylalanine (fMLP) released by bacteria. The receptors are linked to G-protein-receptor signaling pathways, which provide “outside-in” signals. These signals induce PMNs to undergo polarization of their cell form. This process results in the formation of a front end (“leading edge”) and a rear end (“uropod”). At the same time, neutrophil integrins are activated for targeted cell (trans)migration (see chapter 4), enabling PMNs to move intra- and extravascularly with their “front edge” in the direction of the higher concentration of the gradient (88).

However, the exact mechanism underlying the navigation in the complex lymphoid or inflammatory target tissues is not yet fully understood (88). Early studies by Foxman et al. assumed that chemotactic migration is based on a multi-stage process (218). An advanced model of this step-by-step migration developed by Heit et al. described the hierarchy of chemo-attractants. PMNs prioritize chemotactic signals by distinguishing “intermediary” (LTB4, CXCL8, and PAF) and “end-target” chemo-attractants (fMLP and C5a) with significantly different intracellular signaling pathways. Thus, PMNs are able to avoid “distraction” in a complex environment of chemo-attractants and move to the lesion site in a targeted manner (219).

## 7 Formation of Chemotactic Gradients in Interaction With the Extracellular Matrix

The original concept of the chemotaxis of cells was described as directional migration heading for a concentration gradient of soluble chemo-attractants. Later, the gradient of chemo-attractants was found to be generally determined by the binding and immobilization of these chemical signals to a substrate. The concept of haptotaxis was introduced, denoting directional cell movement induced by a gradient of structure-bound adhesion sites or signal molecules (216, 220–222).

Within the vascular compartment, chemo-attractants are immobilized by glycosaminoglycans (GAGs) or heparan sulfate, mainly on the luminal membrane of endothelial cells (223–227). Outside vessels, chemo-attractants can bind to the ECM, thus directing the migration of neutrophils to lesion foci (216). On their migration path, PMNs come into contact with two different basic forms of the ECM: On the one hand, with basement membranes consisting of thin networks of tightly interconnected glycoproteins, and, on the other hand, by meeting loose fibril-like interstitial matrices after transmigration (198). As described above, the basement membrane consists of the four main components collagen IV, laminin, nidogen, and heparan sulfate as well as of the proteoglycan perlecan. With the exception of the CNS, the interstitial matrix in most tissues is composed of fibrillae that mainly contain collagen of types I, III, V, and XI (198). In addition, specialized ECM structures exist that combine the properties of both basement membrane and interstitial matrix. These structures form the reticular fiber network of the secondary lymphatic organs and share properties with the provisional matrix formed at injury sites (198). The negative charge of many ECM molecules, in particular of proteoglycans, and the large surface they occupy in the tissue offers a large potential for interactions with other charged molecules such as chemokines (198). Thus, chemo-attractants can bind to the proteoglycans of the ECM, thereby directing the migration of neutrophils to lesion foci (216).

## 8 The Role of Microtubules and the Microtubule Organizing Center in the Migration of Neutrophils

As postmitotic cells, neutrophils are not able to undergo mitosis. To run their function in host defense, PMNs are not reliant on the mitotic machinery. The advantage of this minor microtubule architecture in combination with the segmented nucleus may enable high cellular flexibility, which facilitates PMNs to migrate more rapidly than other leukocytes and to infiltrate many different and even dense tissues because of their high morphological dynamics (228). Microtubules (MTs) are known to be substantially involved in intracellular transport. However, the role of MTs in chemotactic PMN migration and neutrophil effector functions is far from being resolved (229).

Whereas resting PMNs contain few MTs, which are gathered in the Microtubule Organizing Center (MTOC, Centrosome) behind the neutrophil multilobular nucleus, PMNs prolongate their MTs within minutes in response to *in vitro* stimulation with chemotactic peptides (such as CXCL-1 or fMLP), as confirmed by Yadav et al. in 2019 (229). Anderson et al. did not report any changes in the number of MTs per neutrophil granulocyte after *in vitro* chemotactic stimulation but a significant increase in the average length of MTs. Thereby, MTs in the direction of migration were lengthened, whereas MTs perpendicular to the direction of migration were shortened (230).

To establish and maintain the necessary cell polarity for amoeboid locomotion (see above), small, rapidly moving cells (as PMNs are) perform actin- and myosin II-dependent reorientation of the MTs array toward their uropod. According to a theory by Eddy et al., such reorientation and compacting accumulation of the MTs into the uropod could make cells more streamlined. Thus, polarization seems to be alleviated, and cell motility could be maximized to two- or three-dimensional matrices. To further elaborate this theory, reorientation of MTs could supply positional information, which would serve to reinforce cell polarity during migration (231).

The change between spontaneous neutrophil locomotion (chemokinesis) and chemotaxis does not seem to involve any changes in the collocation of the microtubule cytoskeleton itself (228). In most migrating cells—including PMNs—, cell polarity is rather characterized by the position of the nuclear-centrosome-axis (NC-axis) in relation to the front-back-axis of the cell. According to Luxton et al., in case of chemotaxing neutrophils in contrast to mesenchymal cells, this NC-axis is oriented in posterior direction. Thus, the neutrophil nucleus is located directly at the leading edge of the cell, and the MTOC is arranged behind the nucleus (232).

Hence, amoeboid PMN migration *in vitro* seems to be consequently characterized by the fact that the nucleus is located ahead and in front of the MTOC in the direction of PMN migration (“nucleus-first-configuration”) (233). Chiplonkar et al. reported that in resting PMNs, the MTOC takes a “predefined” apical location, and only upon chemotactic stimulation *in vitro* do they translocate to a newly defined basal location, if microtubules are intact (234). Anderson et al. reported that MTs spread almost exclusively from a single MTOC after stimulation. In contrast, Schliwa et al. described the transient separation of the centrosome into two single centrioles surrounded by an aster of MTs after PMN stimulation. Schliwa et al. further explicated that 10% of the cells with separated centrosome had a third centriole free aster consisting of microtubules with compactly accumulated seeds (230, 235).

To be able to choose the path of least resistance during migration (see above), PMNs ‘palpate’ their immediate environment. Renkawitz et al. accounted the nucleus-first-configuration as a type of “measure instrument”, possibly enabling the differentiation of the extent of PMNs surrounding pores. In this way, the bulkiest part of the neutrophil, the nucleus, is used as a mechanical gauge and acts as a selector of the migratory direction (233).

In the context of the observed close proximity between nucleus and MTOC and the proof that polarization is determined by the MTOC in other cellular systems, Renkawitz et al. made this assumption as a conclusion of their migration experiments. In these *in vitro* experiments, the MTOC predominantly located itself in between nuclear lobes when cells moved through straight channels. At narrow pores (“decision points”), the neutrophil nucleus unfolded; initially, one nuclear lobe passed preferably the largest pore, before the MTOC and the other nuclear lobes ultimately followed. The MTOC quasi specified the nuclear lobe, resulting in the choice of direction (233). Renkawitz et al. also made the observation that after the nucleus and the attached MTOC had completely overcome the largest pore, cytoplasmatic protuberances located in smaller pores were retightened. This step was coordinated by dynamic MTs, whereby migrating cells loose integrity and fragments in fluid cytoplasmatic pieces in the case of MT rupture (233).

In 2019, a study by Yadav et al. showed that drug-controlled suppression of MT polymerization, which in turn was triggered by chemotactic peptides (for instance, CXCL1 and fMLP), inhibited neutrophil chemotactic migration *in vitro*. This suppression disabled CXCL1- and fMLP-triggered elastase-dependent neutrophil traverse through collagen I hurdles (229). Interestingly, CXCL1-regulated transendothelial migration did not depend on MT polymerization *in vitro*, since the break of existing or *de novo* generated MTs did neither impair protrusion not squeezing through IL-1β stimulated endothelium *in vitro* (229).

Despite the *in vitro* findings described above, we still do not know how microtubules regulate PMN migration *in vivo* (236). In contrast to *in vitro* studies, an *in vivo* zebrafish study by Yoo et al. showed the MTOC in migrating PMNs in front of the nucleus. MT depolymerization inhibited the activity of polarized Phosphoinositol-3-Kinase (PI3K) at the leading cell edge and activated fast PI3K-independent motility. MTs seem to exert their effects on neutrophil polarity and motility *in vivo*, at least partly, *via* negative regulation of both Rho- and Rac-activity (236).

In view of the discrepancies between the *in vitro* und *in vivo* findings, the current state of research is as follows: *de novo* chemoattractant-triggered MT polymerization seems to be the key to neutrophil chemotaxis and elastase-dependent infiltration into tissue but does not seem to be responsible for chemotactic overcoming of the inflammatory endothelial barrier (229).

To conclude, further experiments are required to uncover the discrepancies between *in vitro* and *in vivo* insights and to gain better knowledge about the true role of MTs in neutrophil chemotactic migration and host defense.

## 9 Bidirectional and Reverse Migration of Neutrophils

Perseverance of neutrophils in tissues may result in tissue damage and chronic inflammation as outlined in chapter 11. Therefore, PMNs must be cleared away from the injury site after fulfilling their duty, whereby such neutrophil clearance from affected tissues is crucial to induce a pro-resolution cascade (as reviewed in (237, 238)). Until a few years ago, the predominating dogma of neutrophil clearance after recruitment to tissue was that PMNs undergo apoptosis before they are cleared by macrophages *via* efferocytosis (see chapter 1) (239, 240).

However, a number of questions regarding neutrophil clearance remain undetermined. In various models of sterile inflammation, PMNs infiltrated tissues and disappeared long before the presence of monocytes. Furthermore, in these models, depletion of monocytes or macrophages did not compromise neutrophil removal (237, 241).

**Figure 5:**
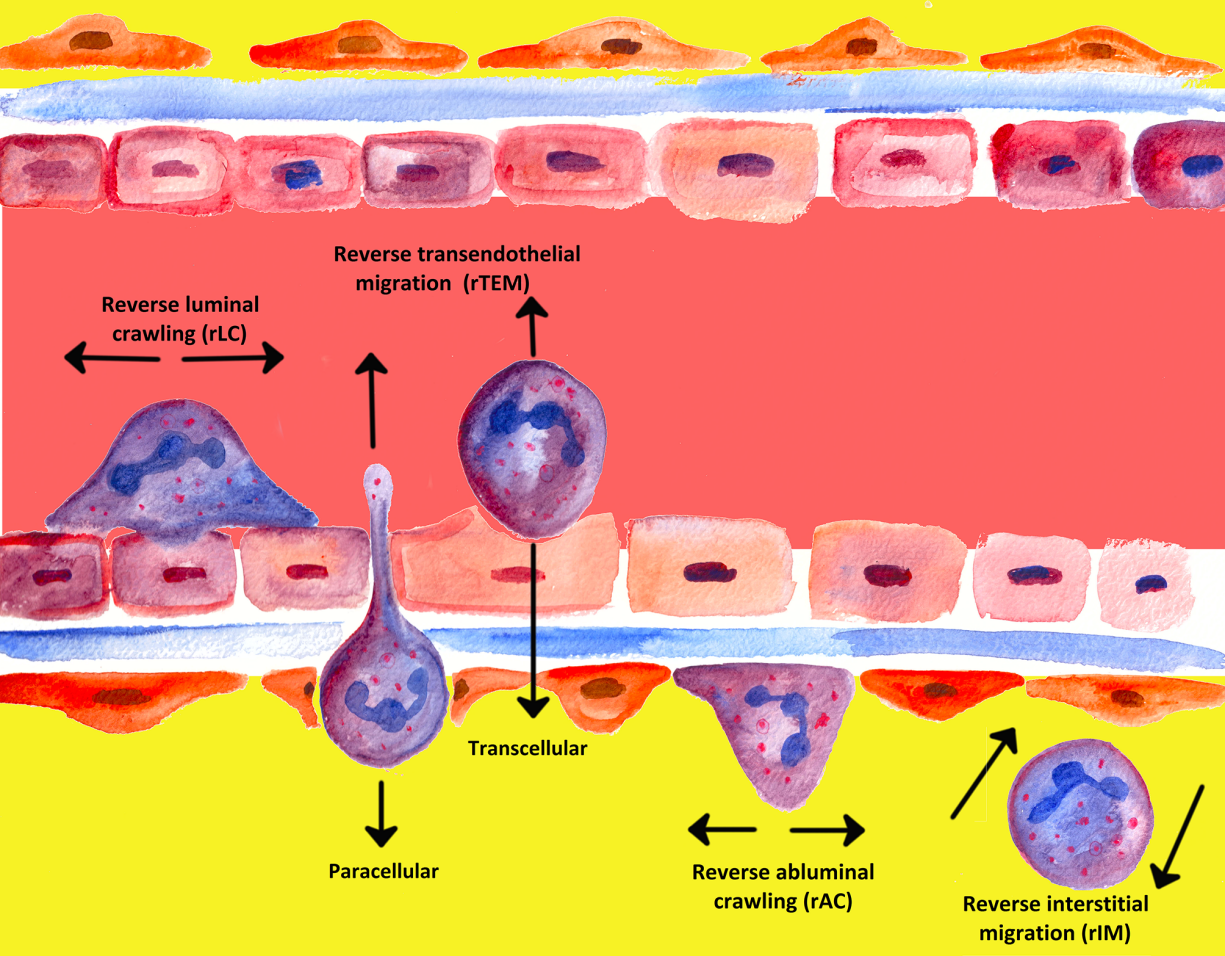
Overview of the different types of migration known for PMNs so far.

An often underappreciated or perhaps ignored issue in the past was whether transmigrated leukocytes can leave inflammatory sites and perhaps even return across the endothelium and re-enter circulation (239). The mechanisms of unidirectional migration of neutrophils through the endothelium into tissues have been extensively investigated; migration from tissues back in the opposite direction, however, has attracted the attention of the scientific world only in recent years (90, 242). In the past two decades, several notable studies have shown that PMNs are able to undergo bi-directional movement and can move in the direction opposite to the direction that was to be expected, a process termed “reverse migration” (243–245). **Figure 5** gives a graphical overview of the different forms of neutrophil migration.

In 1997, Hughes et al. were the first to report in a rat model of glomerular capillary injury that neutrophils can migrate bi-directionally during inflammation (242, 246). Since then, various types of neutrophil reverse migration have been described (243, 245). As outlined by Nourshargh et al., a number of different modes of reverse migration are assumed to (co−)exist, each with its own signal mechanisms and subsequent cell effects (239, 245).

Meanwhile, different denotations have been introduced in the literature for various phenomena of reverse migration, depending on the site. Neutrophil transmigration through endothelial layers in abluminal to luminal direction was denoted as reverse transendothelial migration (rTEM), whereby migration of neutrophils away from inflammatory foci in interstitial tissues was termed reverse interstitial migration (rIM). Besides, reverse abluminal crawling (rAC) constitutes reverse migration of neutrophils in pericyte layers (104, 243–245, 247–249).

The first *in vivo* evidence of reverse transmigration was provided by Mathias et al. in 2006, who demonstrated neutrophils migrating away from a wound back to the vasculature (237, 248). The authors used a genetically engineered zebrafish model, in which neutrophils could be observed by real-time visualization within larvae (239, 248). In the zebrafish model, as many as 80% of PMNs recruited to the injured site migrated back towards the vasculature, whereby some of the cells went even back to circulation; merely 3% of invading neutrophils underwent apoptosis at the site of injury (239, 250, 251). Nevertheless, in zebrafish models, the outcome of PMNs returning to the endothelium has not been fully clarified yet (239).

In 2006, bidirectional movement of human neutrophils through endothelial monolayers was detected by Buckley et al. (252). The authors also described PMNs that displayed reverse transmigration had an altered cell surface phenotype, in which they expressed high levels of ICAM-1 and downregulated expression of the chemokine receptor CXCR1. In this context, it is striking that patients with systemic inflammation show increased levels of this PMN population (ICAM-1^high^/CXCR1^low^) in peripheral blood (239, 252).

In 2011, neutrophil reverse transmigration was also live-imaged in ischemia/reperfusion injury in mouse models (244, 253). Conducting confocal intravital microscopy in mice, Woodfin et al. observed that nearly 10% of transendothelial migration events were reversely migrating PMNs. This finding differed considerably from observations in *in vivo* zebrafish experiments, in which almost all wound responsive neutrophils had migrated reversely (244, 248, 254).

Tharp et al. found that increased levels of cytokine-induced neutrophil chemoattractant-1 [CINC-1, a rat orthologue of human CXCL-8] ultimately result in PMN movement in opposite direction towards venular walls, implicating the concentration of chemo-attractants in one of the major determinants for rTEM regulation (242, 255). Indeed, the process of rTEM seems to depend on the capability of PMNs degrading the junctional adhesion molecule C (JAM-C) by proteolysis (237, 244). As shown by Bradfield et al., JAM-C regulates the unidirectional migration of leucocytes and is ubiquitously expressed on endothelial cells (133, 256). Due to the fact that blockade or genetic deletion of endothelial JAM-C increased neutrophil rTEM, JAM-C was considered an important regulator of rTEM by Woodfin et al. and Zindel et al. (244, 257).

Furthermore, Colom et al. showed that neutrophil elastase was essential for promoting TEM by degrading JAM-C in mice (237, 258). Moreover, LTB4 also seems to influence the regulation of rTEM *via* JAM-C because the application of LTB4, which was observed to enhance the degradation of JAM-C between endothelial cells, increased rTEM in mice. Conversely, in mice pretreated with an LTB4 receptor antagonist, JAM-C expression persisted and neutrophil transmigration decreased (239, 258).

In 2014, Tauzin et al. described the interaction of macrophages with PMN-stimulated neutrophil reverse migration *via* redox-Src family kinase (SFK) signaling, which mediates migration in neutrophils in response to oxidative stress as a redox sensing element (239, 242, 250). Thus, SFK signaling may remove invaded neutrophils to help mitigate neutrophil-mediated inflammation of wounds in zebrafish (242). Neutrophils have been shown to not necessarily require contact with macrophages or monocytes to set up reverse migration. Nevertheless, in the absence of macrophages, the number of recruited neutrophils undergoing reverse migration was significantly decreased (239).

Another factor influencing neutrophil clearance from the site of injury is (de-)stabilization of hypoxia-inducible factor-1a (HIF-1a). Elks et al. demonstrated delayed neutrophil clearance as a consequence of genetic or pharmacologic stabilization of HIF-1a activity. Furthermore, HIF-1a supported inflammation by decelerating neutrophil apoptosis through inhibiting prolyl hydroxylase activity. Burn et al. did not view this decrease in reverse migration as a consequence of overall reduced migration but rather as a change in directionality. This view lead to the suggestion that signaling pathways exist that normally drive PMNs away from the site of initial recruitment (239, 251).

In 2017, Wang et al. described a neutrophil reverse migration cascade from the interstitium backwards using a model of focal hepatic sterile injury (237, 238, 257). The authors observed that PMNs initially performed important repair functions in the interstitial space before migrating back to the bloodstream, whereby PMNs at the injury border showed directional movement away from the lesion (237, 238). After PMNs had entered the bloodstream, they stopped in the lung capillaries, in which CXCR4 was upregulated, which in turn enabled the PMNs to ultimately return to the bone marrow. This process was followed by neutrophil apoptosis and clearance (237, 238, 257). Interestingly, mice deficient in cathepsin C (and thereby unable to activate several proteases) showed normal numbers of neutrophils migrating to the site of injury but fewer neutrophils leaving the lesion, which disrupted the normal revascularization process (237, 238).

Strikingly, CXCR4^high^ neutrophils (“aged neutrophils”) performed reinforced NET formation under inflammatory conditions as asserted by Zhang et al. (259). Moreover, rTEM neutrophils (with phenotype ICAM-1^high^) showed enhanced ability to produce ROS, which in turn is required for NET production (242, 252, 260). These observations led to the assumption that rTEM neutrophils tend to exhibit exceeding NET formation, which—apart from killing invading pathogens—may have negative effects such as tissue injury or disproportionate coagulation during inflammation (242, 260–262).

In 2019, a study on patients with acute ischemic stroke by Weisenburger-Lile et al. determined an increased percentage of neutrophils with a reverse transendothelial migration (ICAM-1^high^CXCR1^low^) phenotype and continuous basal hyperactivation of circulating neutrophils. Importantly, these neutrophil alterations were associated with the clinical severity of the stroke (263). Moreover, Lohri et al. showed that medical interventions can also affect human rTEM neutrophils: After adjuvant chemotherapy in patients with breast cancer, the number of reverse transmigrating (ICAM-1^high^/CXCR1^low^) human neutrophils had decreased significantly (264). These studies highlight not only the diversity of diseases and treatments affecting human rTEM neutrophils but also contribute to the *in vivo* importance of reversely migrating neutrophils and outline the desideratum for a better understanding of proceedings involving reverse neutrophil migration.

On the one hand, reverse migration of neutrophils leads to PMN removing from the lesion site and resolution of local inflammation. On the other hand, reversely migrating neutrophils that re-enter the bloodstream may disperse into different parts of the body by circulation. Taking this hypothesis further, reversely migrating PMNs may transmigrate into other—initially non-inflammatory—organs again, thus contributing to accessory organ injuries and systemic inflammation (242).

Indeed, Yoo et al. observed in a zebrafish model that reversely transmigrating PMNs tended to distribute in tissues throughout the body (242, 250). Similarly, Woodfin et al. found PMNs with phenotype ICAM-1^high^ within pulmonary vasculature after lower-limb ischemic/reperfusion injury in mice. Because of a significant association between the frequency of ICAM­1^high^ neutrophils in pulmonary vasculature of ischemic/reperfusion stimulated mice and the extent of lung inflammation, Woodfin et al. assumed an association of neutrophil rTEM with inflammation in a second organ (242, 244). According to Colom et al., increased JAM-C levels in plasma (as an indirect marker of neutrophil rTEM) correlated significantly with consecutive severity of multiple organ failure in trauma patients (242, 258). Based on these observations, Colom et al. stated that tissue-experienced neutrophils returning to circulation may contribute to propagated systemic inflammation (213, 250, 258).

However, the idea that reverse PMN transmigration promote systemic inflammation after an episode of localized tissue inflammation is controversial. Downregulation of the chemokine receptor CXCR1 (CXCR1^low^-phenotype) and thus the inability of reversely transmigrating PMNs to transmigrate again across inflamed endothelium make it seem unlikely that such PMNs have the capability to reinfiltrate tissue at inflammatory sites (242, 252). Moreover, it is hardly possible to identify rTEM neutrophils by means of upregulated ICAM-1 because ICAM−1 is also upregulated after long-term PMN stimulation by bacterial lipopolysaccharide or cytokines such as TNF- α, as shown by Wang et al. (242, 265).

Previous research mostly focused on the process of reverse migration as a whole (sometimes by reason of investigation methods); in the past few years, however, more attention has been paid to distinguish the different sections of reverse neutrophil migration. Recently, the importance of distinguishing rTEM from reverse interstitial migration (rIM) has been underlined by Nourshargh et al. (245). In contrast to rTEM, rIM constitutes a relatively new field of investigation, which describes movements away from the foci of inflammation within tissues, whereby rIM does not necessarily involve re-entry into circulation *via* the endothelium (239, 245).

So far, a possible connection between these two modes of reverse locomotion has not been examined. The two modes may exist as two separated and autonomous phenomena. Yet, rTEM may also be the continuation of rIM so that PMNs moving from inflammatory foci within tissues (rIM) are able to undergo rTEM after rIM (239). Besides, another purpose of rIM may be the transport of captured antigens to lymph nodes for the initiation of adaptive immune responses as contemplated by Nicolás-Ávila et al. and Maletto et al. (213, 266).

In conclusion, many questions in the field of neutrophil reverse migration remain unanswered. Although recent studies have indicated that neutrophil reverse migration can be physiological as well as pathological, the true (patho-)physiological role of neutrophil reverse migration has not yet been fully elucidated (239, 243). The question which mechanisms and signals are required for establishing reverse migration versus apoptosis also needs to be further investigated (237). Nonetheless, it is noteworthy at this point that both, reverse migration and efferocytosis of PMNs, are not mutually exclusive; in fact, both processes may be necessary for appropriate resolution of tissue inflammation (257).

Even though a few phenotypic markers of rTEM neutrophils have been identified, the specific molecular mechanisms underlying neutrophil reverse migration are far from being completely understood (242). Ultimately, it is indispensable for neutrophils to be removed from the lesion site either by apoptosis or by reverse migration, since the failure to remove neutrophils may lead to disrepair and chronic inflammation (237).

## 10 The Immune Effects of Neutrophils at the Site of Action

**Figure 6:**
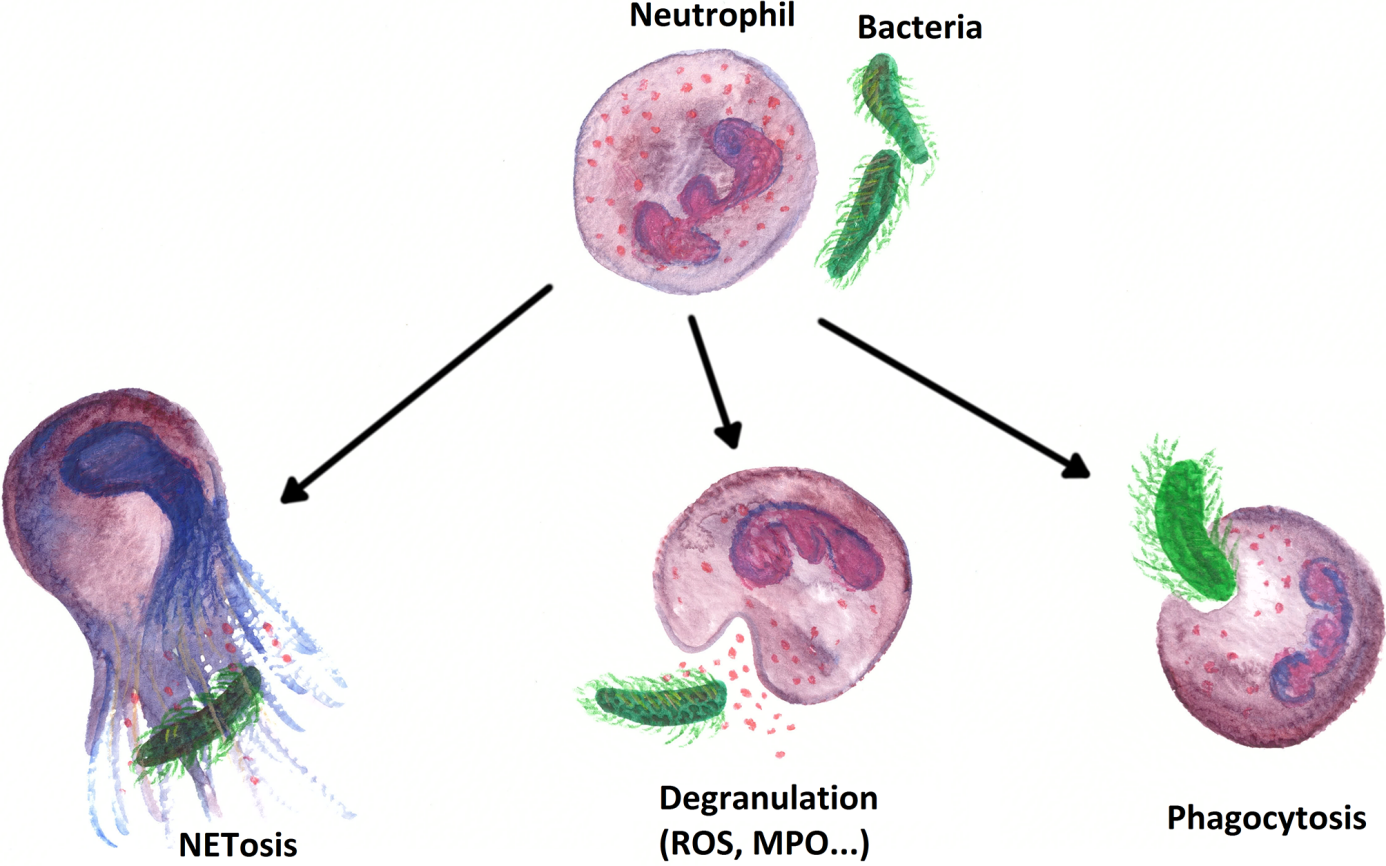
Overview of the most important immune effects PMNs perform within the first line defense of the innate immune system.

After arriving at the sites of action, PMNs have different first line immune defense strategies at their disposal (see **Figure 6**). First, PMNs ensue a form of receptor-mediated endocytosis termed phagocytosis (267). The two main targets of elimination by phagocytosis are foreign particles (pathogens) and “altered self cells”, whereby the term “altered self cell” typically corresponds to apoptotic and necrotic (host-)cells (267–269). In wounds, PMNs also remove dead tissue by phagocytosis, thus preparing the wound for the formation and deposition of new tissue (270). PMNs are professional phagocytes. A single PMN can kill up to 50 individual bacteria. Moreover, neutrophil phagocytosis is a rapid process, which can be completed in just a few seconds (267, 271).

Neutrophil phagocytosis involves a diversity of receptors and starts with the recognition of the target, namely the binding of a phagocytic receptor to its correspondent ligand (267, 269). Receptors on PMN surfaces are capable of recognizing phagocytic determinants that are intrinsic to pathogens (i.e., PAMPs) and classified by the C-type lectins Dectin-1 (which binds to β-glucan) and Dectin-2 (which is able to bind to a variety of ligands on the surface of mycobacteria, fungi, and even cancer cells) (267, 272–274). Receptors that detect eat-me-signals of “altered self cells” bind directly to phosphatidylserine (PS) or PS-binding bridging proteins, altered sugars (recognized by lectins), or thrombospondin (267, 275).

Although recognition of PAMPs can trigger phagocytosis, microbial engulfment is at its optimum when targets are “marked” as foreign cells by being coated with distinct serum components that can be detected by effective phagocytic receptors. This process of “labeling” certain microorganisms by antibodies and the complement system are known as opsonization (267). The most important opsonins in serum are immunoglobulins and certain components of the complement cascade (reviewed in (267)); opsonins are recognized by both Fc receptors (FcRs) and complement receptors (CRs) (reviewed in (276)) (267, 277).

The binding of multivalent ligands to the surface of the target leads to the clustering of receptors on the PMN and—after various intermediate steps—to the recruitment of GTPases of the Rho family (278, 279).

The following signal cascade results in the actin-dependent formation of a phagocytic cup and the elongation of pseudopodia around the ligand. Finally, the target is ingested into a vacuole—the phagosome—that is completely internalized into the neutrophil cell. The phagosome undergoes extensive remodeling to increase its hostile mechanisms against pathogenic particles. This process is known as maturation, by which internalized particles are moved into a series of soaring acidified membrane-bound structures, culminating in particle degradation and elimination of the ingested microorganisms (267, 280). Although some bacteria have developed strategies to survive phagocytosis, it should be mentioned at this point that phagocytosis does not inevitably lead to the destruction of all microbes. Staphylococcus aureus, for example, impedes phagocytosis on itself by complement inhibitors but can also escape intracellular destruction by enzymes such as superoxide dismutase (281, 282). Nevertheless, neutrophil phagocytosis is an effective first line defense within the innate immune system.

**Video 1** was provided by Franz Reichelt (Laboratory of anesthesiology, University Medical Center Regensburg) and shows the process of phagocytosis as described above by means of *in vitro* phagocytosis of Escherichia coli (stained red) by PMN. The experimental assay shown is a chemotactic experiment according to Doblinger et al.; in this experiment, an fMLP-chemotactic gradient was built in a 3D collagen matrix, in which PMNs were embedded, enabling them to move and mediate their immune effects along the gradient (283).

In addition to the phagocytosis of pathogens, PMNs use two fundamentally different mechanisms for the defense against infectious pathogens: oxygen-dependent and oxygen-independent mechanisms (284, 285).

As oxygen-dependent mechanism, the formation of reactive oxygen species (ROS) should be mentioned in particular (285). In the context of a process termed “respiratory burst reaction”, phagocytizing PMNs show a strong increase in their oxygen consumption. This increase is caused by the NADPH-dependent production of superoxide anions ${\text{O}}_{2}^{-}$ , which are the trigger that leads to the formation of ROS, *i.e.* to the formation of hydrogen peroxide (H_2_O_2_), hydroxyl radical (OH•), and hypochlorous acid (HOCl). These acids contribute to the destruction of bacteria (286, 287). In **Video 2**, the process of ROS production is illustrated showing fluorescence images of an *in vitro* chemotaxis experiment with human PMNs (199): Human PMNs were embedded in a type I collagen matrix and exposed to an fMLP gradient. ROS production was visualized using 1,2,3-dihydrorhodamine (DHR). The red glowing signal around the cells indicates an ongoing ROS production in the videos.

As oxygen-independent mechanism, the degranulation of histologically visible granules is of importance because of its release of lytic enzymes and bactericidal peptides (284). Cytoplasmic granules are characteristic for neutrophils (which belong to the granulocyte family) and instrumental in microbicidal response. These granules can be subdivided into three dissimilar classes based on the contents of their matrix and their integral membrane proteins: azurophilic (primary) granules, specific (secondary) granules, and gelatinase (tertiary) granules (267, 281). Primary granules contain antimicrobial substances, such as lytic enzymes and antimicrobial peptides, and include defensins and myeloperoxidase (MPO). Secondary granules contain phagocytic receptors (e.g., Fc receptors and CRs; see above) and the NADPH oxidase complex (see above). Tertiary granules contain receptors and enzymes that degrade ECM to facilitate the extravasation process and the migration of neutrophils to the site of inflammation (see *The Process of Extravasation*) (267, 288). Taken together, degranulation results in the release of lytic enzymes and bactericidal peptides, procuring an effective host defense against microbial pathogens.

However, one granule component that plays a special role in oxygen-independent defense mechanisms is the enzyme myeloperoxidase (MPO). In the presence of H_2_O_2_ and chloride anions (Cl^-^), MPO catalyzes the formation of reactive oxygen intermediates including HOCl, which destroys cell membranes and cell walls. Besides the antimicrobial effect of the MPO/HOCl system, MPO has proved to be a local mediator of tissue damage and the resulting inflammation in various inflammatory diseases (289). **Video 3** shows the release of neutrophil MPO in an chemotaxis experiment, where PMN were embedded in a type I collagen matrix and exposed to an fMLP-gradient. In this experiment MPO was made visible by ANTI-MPO-APC anti-body staining, so that the green signal in the video near the cells indicates just released MPO (199).

In 2004, Brinkmann et al. (290) described another, previously unknown ability of PMNs: At the end of a cytolytic process, the nucleus of PMNs is released as a net-like DNA structure into the extracellular space (284). These neutrophil extracellular traps (NETs) have histones and cationic peptides on their surface (284). Once released, NETs can surround, immobilize, and finally kill both bacteria and fungi. The phenomenon of NET release mainly occurs in inflammation foci and is referred to as NETosis (290). In **Video 4** NETosis was visualized in an *in-vitro*-chemotaxis experiment with human PMNs (199). The PMNs were embedded in a type III collagen Matrix and exposed to an fMLP gradient. NETosis was visualized was assessed with 4´,6-diamidino-2-phenylindole. In the beginning, PMNs migrate along an fMLP gradient within the matrix, whereby they produce ROS (visualized by DHR red signal). With increasing experimental time, neutrophil migration stopped and the cells underwent NETosis. Thereby, the blue signal in the videos indicates PMNs undergoing NETosis.

Three models of NETosis have been described so far.

First, the best described model is suicidal NETosis with a duration of 2–4 h (291). NETosis begins with the activation of neutrophils through the recognition of stimuli (PMA or fMLP, among others), leading PMNs to stimulate the NADPH oxidase complex through protein kinase C (PKC), Raf, MERK, and MAPK/ERK signaling (292, 293). Furthermore, the activation of peptidyl arginine deiminase 4 (PAD4)-dependent citrullination of histones induces the decondensation of chromatin (292, 294–296). Suicidal NETosis is dependent on ROS for the disintegration of the nuclear membrane and for histone citrullination by PAD4 (295–297). Suicidal NETosis also depends on elastase and MPO transport from granules to the nucleus (298). Ultimately, pores in the ruptured plasma membrane allow the liberation of NETs, leading to cell death and the loss of viable cell functions (292, 294, 299–301).

The second model is vital *NETosis*, during which PMNs release NETs without destructing the plasma membrane or the nucleus. This type of NETosis lasts about 5–60 min and consists of the release of nuclear DNA through nuclear shell growth and vesicle release, decondensation of the nucleus, and nuclear shell disruption (291, 302–304). Vital NETosis is promoted by activation of TLRs and complement receptor for C3 protein (292, 305–307). Moreover, interaction between platelet glycoprotein Ib with β2-integrin may induce NET formation by activating ERK, PI3K, and Src kinases (292, 308). Neutrophils undergoing vital NETosis are still able to run phagocytosis with preservation of chemotaxis (294, 309, 310), allowing the coexistence of NET forming and conventional host defense (292, 304).

Finally, a third type of NETosis was described by Yousefi et al. in 2009. In this subtype of vital NETosis, which is dependent on ROS, mitochondrial DNA is released instead of nuclear DNA. After recognition of complement C5a or lipopolysaccharide (LPS), mitochondrial NETs are released from 80% of neutrophils within 15 min (292, 311).

Even though great progress has already been made in this field in the past decade, the cellular mechanisms that mediate neutrophil NET release are still not fully explored (304).

**Figure 7:**
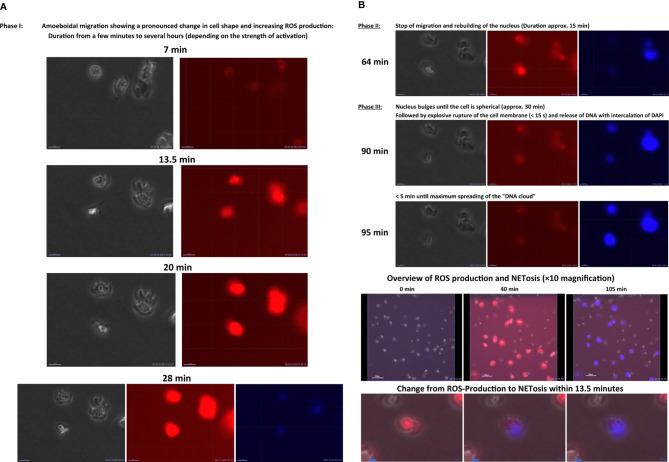
**(A, B)** Graphical presentation of the chronological sequence of the neutrophil immune effects ROS production and NETosis. Fluorescence images of an *in vitro* chemotaxis experiment with human PMNs: The cells were embedded in a type I collagen matrix and exposed to an fMLP gradient. ROS production was visualized using dihydrorhodamine 123 (red). NETosis was assessed with 4´,6-diamidino-2-phenylindole (DAPI, blue). The time points in the headlines of the images refer to the time of first gel contact. Overview of the sequence of neutrophil immune effects ROS production and NETosis in in-vitro-chemotaxix experiment (x40 magnification).

The chronological sequence of neutrophil immune effects (migration, ROS production, MPO release and NETosis) is recognized in the current scientific literature and reflects the fact that ROS und MPO are released through degranulation first, whereas NETosis occurs last (260, 283, 312). While **Figures 7A, B** illustrates this sequence of immune function using images, **Video 5** shows the processes using film techniques. In both media an *in vitro* chemotaxis experiment (like those in **Videos 2–4**) is presented in which PMNs were embedded in a matrix of type I collagen and exposed to an fMLP gradient. First, PMNs migrate along the gradient, whereby they produce ROS (red signal). With increasing experimental duration, migration decreases and the cells undergo NETosis one after another.

The relationship between neutrophil phagocytosis and NETosis is still controversially discussed. Some literature reports suggest that the two immune effects cannot coexist within one cell at the same time. Some researchers, such as Manfredi et al. and Branzk. et al., postulated that—after phagocytosis—PMNs can no longer perform NETosis and, conversely, PMNs perform NETosis if they are unable to phagocytose the pathogen (for example because of its size) (313, 314). This theory is based on results indicating that the presence of MPO and neutrophil elastase in the cytosol is a prerequisite of NETosis. These enzymes would be captured during phagocytosis and phagosome maturation; therefore, they would not be available in the cytosol and thus at the nucleus for NETosis any longer (314–316). Manfredi et al. concluded that neutrophils encountering a pathogen make an irrevocable decision to either phagocytose or to form NETs (313).

However, there are doubts if the only option for neutrophils is truly dichotomous and not a consecutive process from phagocyosis to NETosis. This assumption is supported by a publication by Ullah et al. showing pneumococcal-induced autophagocytosis as a promoter of NETosis (317). Furthermore, Pelletier et al. demonstrated by means of flow cytometry that PMNs, which have already phagocytosed subsequently went into NETosis (318). A matter of interest in this context is the observation that after the phagocytosis of certain bacteria with evolved mechanisms for escaping intracellular death (such as E. coli or S. aureus), PMNs released the previously internalized bacteria just before neutrophil cell dead was induced (319–323).

The above process is shown in **Video 6**, which was provided by Franz Reichelt (Laboratory of anesthesiology, UMC Regensburg). The experimental setup in **Video 6** is equal to that in **Video 1**. The video shows a neutrophil cell that phagocytosed 6–8 individual bacteria of E. coli *in vitro*. Having obviously already committed to the defense function of phagocytosis, the neutrophil cell releases the bacteria and still undergoes NETosis afterwards. As the bacteria are shown to actively move in the video, they appear to be alive after their release. Thus, the bacteria seem to be still able to replicate and be capable of causing harm to the host (321, 323).

The inconsistencies just reflect the need for further elucidative work in the area of granulocyte functionality in contact with bacteria.

In this context, we introduce another (rather unknown) neutrophil immune function. In the presence of bacteria, PMNs develop dynamic, thin, and very long membrane tubules that are able to catch pathogens. These protrusions of the cytoskeleton and the cell membrane are referred to as tubulovesicular extensions (TVE), protrusions, or cytonemes (324, 325). Galkina et al. showed that cytonemes are able to bind and aggregate bacteria at a distance by telescopic exocytosis to release bactericidal molecules directly at the bound pathogen but not in their own vicinity (326, 327). The outstanding length of cytonemes—which can reach several cell diameters in length—allows PMNs to secrete targeted aggressive bactericides over a long distance without diluting or injuring the surrounding tissues (328). Cytonemes participate in neutrophil migration in an actin-dependent manner but seem to be dispensable for cell locomotion (325, 329).

Furthermore, cytonemes have been shown to execute long-range adhesion and binding objects for phagocytosis, such as serum-opsonized zymosan particles and erythrocytes (155).

In addition, Kornberg et al. suggested in 2014 that the main task of cytonemes was intercellular communication by means of receptor-ligand-interactions. By allowing signals to be transmitted from a source cell to target cells in a selective manner over a range of distances, cytonemes have recently emerged as a means of communication between cells in a highly specific manner (330–333). In recent years, the influence on the formation of cytonemes has been investigated. Cytonemes are assumed to be influenced by certain microbial substances such as the alkaloid staurosporine, Adenosine-A_3_ receptors agonists, and the presence of nitric oxide, which also seems to play a crucial role in the regulation of cytonemes (155, 334, 335).

Although or just because they are occasionally rather difficult to see with light microscopy due to their extremely small diameter, cytonemes are a wonderful example of the fascinating range of functions and peculiarities of PMNs.

**Video 7** (provided by Franz Reichelt, UMC Regensburg) shows the formation of neutrophil cytonemes in an *in vitro* chemotaxis experiment, in which PMNs were embedded in a type I collagen matrix, exposed to an fMLP gradient, and placed next to E. coli. Phase-contrast exposure shows some kind of cell filament formation with long, thin protrusions suddenly spreading from the cell followed by their prompt regress in the middle of the screen. The process takes about 10 minutes and includes protrusions up to 110 µm in length. In this video, it is also worth to pay attention to the cell touched by the protrusions, which is followed by NETosis as indicated by blue DAPI signaling. In addition, it looks like cytonemes are also spreading from this second cell.

To sum up, cell migration, phagocytosis, oxidative burst, degranulation, and NETosis are some of the most important functional responses that enable PMNs to fulfil their tasks in immune defense (336).

## 11 Importance of a Balanced Immune Response of Neutrophils

The importance of a functioning PMN immune response can be above all seen in the severe courses of disease in which a disordered PMN immune response can lead to life-threatening infections, such as chronic granulomatosis, leukocyte adhesion deficiency, or all forms of neutropenia (337–339).

However, the mechanisms used by PMNs to kill microorganisms also have the potential to injure healthy tissue. Thus, excessive PMN response has a negative effect on the course of certain inflammatory diseases, such as acute respiratory distress syndrome (ARDS), cerebral apoplexy, acute coronary syndrome, or sepsis (340–342). Hereunto, it is important to point out that neutrophil NETs could have problematic effects under certain conditions, as most recently shown by studies of SARS-CoV-2 (343, 344). Furthermore, a number of autoimmune diseases are not directly caused by malfunctioning PMNs but indirectly by the significant contribution of PMNs to the pathogenesis of these diseases (345). Thus, an important influence, *inter alia*, on the autoimmune diseases systemic lupus erythematodes (SLE), rheumatoid arthritis (RA), or pyoderma gangrenosum (PG) is attributed to dysregulated PMN immune response (312, 346, 347). PMNs even appear to be involved in the pathogenesis of degenerative CNS diseases such as Alzheimer’s disease or multiple sclerosis (348–350). In the context of dysregulated PMN migration, it is worth mentioning that, in Alzheimer’s disease, LFA-1-mediated neutrophil transmigration through the blood-brain-barrier (BBB) may promote neutrophil inflammation within the brain together with amyloid deposits, leading to far-flung neutrophil-dependent CNS damage (348, 350). Besides autoimmune and degenerative diseases, neutrophil defense mechanisms also seem to have a destructive effect on the integrity of the BBB in infectious diseases. Thus, diseases with lesion sites primarily outside the CNS may suddenly involve the CNS, as observed in the cerebral manifestation of malaria (350, 351).

The important role of neutrophils in innate immunity, together with their tendency to cause tissue damage, requires the balanced and strict control of PMN activity (7).

## 12 Neutrophil Behavior in Cancer Environment and Tumor Tissue

Cancer is a chronic disease, which critically relies on the interplay of tumor cells with their supporting environment. Cancer presents with inflammation, and inflammatory response is an important factor for the development of tumors (213, 352). Compared to other immune cells, neutrophils have traditionally received little attention in this field, partly because their limited lifespan seems to tergiversate with the chronic nature of cancer. Experimental evidence generated in the past decade, however, supports a causal role for neutrophils in malignant transformation, tumor progression, antitumoral immunity, and angiogenesis (213, 353, 354).

It becomes more and more evident that tumor-associated neutrophils (TANs) and their myeloid precursors (peripheral neutrophils and granulocytic Myeloid Derived Suppressor Cells [G-MDSCs]) in bone marrow, spleen, and blood have an important role in cancer biology (353, 355). Although it is unlikely that immune suppression is their only biological function (as noted by Coffelt et al.), the term G-MDSC is used to indicate the immunosuppressive pro-tumoral properties of this heterogeneous group of cells of myeloid origin, including neutrophils (212, 356).

A transcriptome study by Fridlender et al. showed that TANs are not “tissue-based G−MDSCs” modulated by the tumor micro-environment (TME) but are a different population of neutrophils from both bone marrow-derived neutrophils and G-MDSCs (353, 355). The spleen is known as the site of TAN precursor localization, from which they physically relocate to tumor stroma, whereby CXCL8 (IL-8) is mainly responsible for the recruitment of TANs (353, 357). The make-up of the myeloid compartment in tumor stroma seems to be determined by the TME rather than by the anatomic site of tumor development or tumor-derived circulating factors (358).

Extravasation from blood into a tumor is a regulated multistep process involving a series of coordinated interactions between PMNs and endothelial cells. This process is partly different from non-tumorous situations. A cytokine-endothelium cross-talk is the first step in the intratumoral accumulation of PMNs (359, 360). Some pro-inflammatory mediators or other factors directly secreted by tumor cells or elicited as downstream mediators by the released cytokine increase the endothelial expression of several leukocyte adhesion and activation molecules. IL-1β and TNF-α induce or up-regulate the expression of endothelial-leukocyte adhesion molecule 1 (ELAM-1), P-selectin, ICAM-1, and vascular cell adhesion molecule-1 (VCAM-1) in endothelial cells, whereas Interferon-γ (IFN- γ) mainly promotes ICAM-1 expression (359, 361–364).

Integrin-mediated adhesion leads to the extravasation of PMNs, which are highly attracted to the tumor site by the macrophage inflammatory protein 2 (MIP-2) binding to the CXCR1 or CXCR2 counter receptor of PMNs. MIP-2 expression was associated with marked recruitment of PMNs, whose accumulation was enhanced by the further release of MIP-2 produced by PMNs themselves in response to the stimulation by TNF-α in the TME (359, 365).

PMNs also accumulate in tumor stroma when IL-10 is present in the TME (359, 366, 367). IL-10 is typically regarded as an anti-inflammatory mediator because it inhibits the release of other interleukins and chemokines (359, 368–370). Furthermore, a distinct adhesion pathway, mediated by CD11b/CD18 up-regulation on activated PMNs, enables these cells to adhere to the vascular endothelium, thus creating a subjacent micro-environment. The subsequent accumulation of neutrophil effector molecules at local concentrations is sufficient to cause endothelial damage and matrix degradation (359, 371).

The role of TANs in tumor progression or eradication and metastasis has been controversially discussed in the literature [reviewed in (253)]. TANs have pro-tumorous properties but may also act as antitumor effector cells (372–374). In the early 2000s, this contradictory role of neutrophils in both tumor suppression and tumor promotion was re-evaluated in terms of the characterization of different types of TANs with polarized N1 (anti-tumorous) or N2 (pro-tumorigenic) phenotypes (356, 373). The contradictory evidence can be partly explained by the high plasticity of neutrophils in response to primary tumors. After the migration into tumor tissues, neutrophils specialize under the direct influence of factors secreted by tumor cells and acquire various phenotypes and functions. This process seem to be controlled by TGFβ in tumor proximity (356, 375). The description of TAN subtypes N1 and N2 illustrates how the TME can influence the phenotype of these cells (356).

Under the influence of TGFβ in the TME, TANs polarize to N2 cells, which are characterized by an expression profile that promotes tumor angiogenesis and metastasis and inhibits antitumor immune response (372, 376–379). In the context of TME, secreted ROS, RNS, and proteases may lead to oxidative damage, thus inducing genetic damage or signaling in pre-tumoral cells, which subsequently results in boosted tumorigenesis (213, 372). During tumor progression, N2 cells become predominantly pro-tumorigenic: The transfer of neutrophil elastase (NE) by N2 cells activates proliferation within tumor cells. The liberation of arginase-1 (ARG-1) suppresses CD8+ T-cell and NK cell responses, and the release of MMP9 activates the vascular endothelial growth factor A (VEGFA) and fibroblast growth factor (FGF2), which support angiogenesis (213). Furthermore, N2 neutrophils are characterized by the high expression of CCL2 and CCL5 chemokines and the ability to inhibit effector T-cell functions (356, 373).

Under TGFβ-inhibiting conditions, neutrophils acquire an antitumor N1 phenotype, which promotes tumor death and inhibits tumor growth (359, 373, 380, 381). N1 neutrophils can be identified by hypersegmented nuclei, increased expression of intercellular adhesion molecule (ICAM) and TNF-α, and the ability to activate CD8^+^ T lymphocytes (356, 372). It is still unclear whether the adhesion of PMNs to tumor cells is necessary to cause injury. However, the ultrastructural studies conducted during the growth and rejection phases of several tumors engineered to release cytokines have shown PMNs to be in close contact with damaged tumor cells (359, 382, 383).

Interestingly, N1 and N2 neutrophils were shown to control the activation status of CD8^+^ T−cells. This interplay seemed to be reciprocal because activated CD8^+^ T-cells also controlled the activation and migration of neutrophils to the TME (372, 384).

A reverse reprogramming effect has been shown to be exerted by interferons (IFN); IFN-γ and the granulocyte macrophage colony-stimulating factor (GM-CSF) induce an anti-tumoral phenotype in human neutrophils; such neutrophils are capable of cross-presenting antigens, which triggers and augments T-cell responses (213, 385).

Clinical evidence also indicates a negative association between the number of TANs and the prognosis for many types of cancer including malignant melanoma, renal carcinoma, colorectal cancer, gastric or pancreatic ductal carcinoma, hepatocellular carcinoma, intrahepatic cholangiocarcinoma, and head and neck cancer (386). Templeton et al. demonstrated a significant correlation between circulating neutrophil counts (respective neutrophil-to-lymphocyte ratios) and the overall survival of patients with solid and hematological tumors (387). Up to now, the neutrophil/lymphocyte ratio is being used as a prognostic factor in colorectal and non-small-cell lung cancers (356, 388, 389). Tumor infiltration by MPO expressing neutrophils was shown to be an independent prognostic biomarker with a favorable prognosis in human breast cancer (390).

Besides prognostic issues, the secretion of MPO by TANs is also important for the recruitment of monocytes and macrophages and the activation of platelets in tumor settings (356, 391). In turn, the combination of platelet count and neutrophil to lymphocyte ratio is considered to be a useful predictor of postoperative survival in patients with colorectal cancer, which shows the close interconnectedness of the different myeolic cells in tumor processes (392).

Although NETosis has also been found in non-microorganism-induced inflammatory environments such as tumors, the precise details of the connection between NETosis and tumor processes are not yet known (393, 394). The limited data available do not provide sufficient evidence to conclusively demonstrate whether TANs actually produce NETs and to outline what signaling pathways are involved in NETosis in the TME. Despite the available knowledge about the relationship between the deposition of NETs and the recruitment of MPO-rich population of neutrophils in tumors, there does not seem to be enough evidence to prove the existence of TAN-specific NETosis (353, 393–395).

The contradictory role of neutrophils in tumor events remains the subject of intense research. However, the detailed mechanisms of the immunosuppressive function of TANs and their exact role in tumor progression are still largely unknown (396). What we do know is that in the absence of pro-inflammatory cytokines such as IL-1β, TNF-α, and GM-CSF, paracrine IL-10 debilitates the early influx of PMNs and permits initial tumor formation by transitorily paralyzing a prompt non-specific antitumor response (359, 366, 397). Recently, a new hypothesis regarding the immunosuppressive ability of TANs has been presented in the literature by Hiramatsu et al., who investigated the mechanisms behind the immunosuppressive ability of TANs in gastric cancer. The authors observed that neutrophils incubated with tumor-tissue-culture supernatants (TTCS) of gastric tumor cells showed upregulation of programmed cell death ligand 1 (PDL−1) expression, a decreased ratio of apoptotic cells, decreased expression of human leukocyte antigen DR (HLA−DR), and diminished levels of H_2_O_2_. Subsequently, Hiramatsu et al. found that neutrophils in non-inflammatory tumor tissue inhibit the proliferation of CD4^+^ T-cells and may form a local immunosuppressive environment through the PD−1/PDL−1 pathway (396).

The programmed cell death protein 1 (PD-1) and PDL-1 is a negative immune checkpoint pathway that inhibits immune responses, whereby upregulation of PD-1 in exhausted T-cells enables cancer cells to evade immune responses (398). Blockade of the PD-1/PD-L1 pathway has been shown to invert immunosuppression and to rehabilitate the function of T-cells in tumor tissues. Currently, immune checkpoint inhibitors are one of the most encouraging cancer immunotherapy strategies (399).

To sum up, the role of TANs is not yet fully clarified. A better understanding of the mechanisms by which PMNs interact with the specific immune system in tumor defense and act to enhance or inhibit tumor growth is essential to provide novel approaches for cancer treatment by promoting antitumor immune responses based on stimulation of neutrophil antitumor capabilities (356, 399).

## 13 Summary

To contribute to a better understanding of the role of neutrophils in the human organism, this review summarized current knowledge about PMN chemotaxis and bidirectional migration and PMN interaction with ECM. We considered the role of neutrophil microtubules in migration and discussed neutrophil behavior in the context of cancer environment and tumor tissue. Despite recent successes in elucidating newly discovered neutrophil properties and functions, many processes are not yet fully clarified and require further research. The aspiration of future studies should therefore be to mimic physiological conditions as closely as possible by refining existing models or by creating new assays. In this way, neutrophil key mechanisms along with signaling pathways can be investigated, enabling the development of effective treatment methods (88).

## Author Contributions

The corresponding author RK gathered the information, did the literature research, summarized the findings, wrote the first, the following drafts and the endversion of the manuscript. RK also created the tables, edited the video material and took care for the visual and added all changes that occurred during the process of writing. MG had the idea for this review, corrected the drafts, gave suggestions about the topics and did the professional correction of the manuscript. All authors contributed to the article and approved the submitted version.

## Conflict of Interest

The authors declare that the research was conducted in the absence of any commercial or financial relationships that could be construed as a potential conflict of interest.

## Publisher’s Note

All claims expressed in this article are solely those of the authors and do not necessarily represent those of their affiliated organizations, or those of the publisher, the editors and the reviewers. Any product that may be evaluated in this article, or claim that may be made by its manufacturer, is not guaranteed or endorsed by the publisher.

## Glossary

**Table:**

APC	Antigen presenting cell
ARDS	Acute Respiratory Distress Syndrome
ARG-1	Arginase 1
BBB	Blood-brain-barrier
BLT1	Leukotriene B4 receptor 1
C5a	Activated complement factor 5
CCL	Chemokine (C-C motif) ligand
CD	Cluster of differentiation
CINC-1	Cytokine-induced neutrophil chemoattractant 1
CNS	Central nerve system
COVID-19	Coronavirus-induced disease 2019
CTLD	C-type lectin domain
CXCL	Chemokine (C-X-C motif) ligand
DAMP	Damage-associated molecular pattern
DAPI	4′,6-Diamidin-2-phenylindol
DHR	1,2,3-Dihydrorhodamin
DIC	Disseminated intravascular coagulation
DNA	Desoxyribonucleic acid
DPEP-1	Dipeptidase 1
EC	Endothelial cell
ECM	Extracellular matrix
ECMO	Extracorporeal membrane oxygenation
ELAM-1	Endothelial-leukocyte adhesion molecule 1
ENA-78	Epithelial-derived neutrophil-activating peptide 78
ERK	Extracellular signal-regulated kinases
ESL-1	E-selectin-ligand-1
FGF2	Fibroblast growth factor 2
fMLP	*N*-formylmethionine-leucyl-phenylalanine
FPR	Formyl peptide receptor
GAG	Glycosaminoglycane
G-CSF	Granulocyte colonization stimulating factor
GlyCAM	Glycosylation-dependent cellular adhesion molecule
GM-CSF	Granulocyte macrophage colony-stimulating factor
G-MDSC	Peripheral neutrophils and granulocytic Myeloid Derived Suppressor Cell
GPC(R)	G-protein-coupled receptor
GRO	Growth-regulated protein
H_2_O_2_	Hydrogen peroxide
HA	Hyaluronic acid
HIF-1a	Hypoxia-inducible factor-1a
HLA–DR	Human leukocyte antigen DR
HOCl	Hypochlorous acid
ICAM	Intercellular adhesion molecule
IFN- γ	Interferon-γ
IL-8	Interleukin 8
JAM	Junctional adhesion molecule
LFA-1	Lymphocyte function-associated antigen 1
LPS	Lipopolysaccharide
LSALT	Synthetic peptide H-LSALTPSPSWLKYKAL-NH
LTB4	Leukotriene B4
MAC-1	Macrophage antigen 1
MAPK	Mitogen-activated protein kinases
MCP-1	Monocyte chemoattractant protein 1
MIF	Macrophage migration inhibitory factor
MIP-2	Macrophage inflammatory protein 2
MMP	Matrix metalloproteinase
MPO	Myeloperoxidase
MT	Microtubule
MTOC	Microtubule Organizing Center
NADPH	Nicotinamide adenine dinucleotide phosphate
NAP-2	Neutrophil-activating peptide 2
NE	Neutrophil elastase
NET	Neutrophil extracellular trap
OH•	Hydroxyl radical
PAD4	Peptidyl arginine deiminase 4
PAF	Platelet activating factor
PAFR	Platelet activating factor receptor
PAMP	Pathogen-associated molecular pattern
PD-1	Programmed cell death protein 1
PDL–1	Programmed cell death ligand 1
PECAM-1	Platelet endothelial cell adhesion molecule 1
PG	Pyoderma gangrenosum
PGP	Proline-glycine-proline
PI3K	Phosphoinositide 3-kinase
PKC	Protein kinase C
PMA	Phorbol 12-myristate 13-acetate
PMN	Polymorphonuclear cell
PRR	Pattern recognition receptors
PSGL-1	P-selectin glycoprotein ligand 1
PTX-3	Pentraxin 3
RA	Rheumatoid arthritis
rAC	Reverse abluminal crawling
RANTES	Chemokine regulated upon activation normal T-cell expressed and secreted
rIM	Reverse interstitial migration
ROS	Reactive oxygen species
rTEM	Reverse transendothelial migration
SDF-1	Stromal cell-derived factor-1
SFK	Redox-Src family kinase signaling
SLE	Systemic lupus erythematodes
SRC	Proto-oncogene tyrosine-protein kinase SRC
TAN	Tumor associated neutrophil
TME	Tumor micro-environment
TNF–α	Tumor necrosis factor α
TTCS	Tumor-tissue-culture-supernatants
VCAM–1	Vascular cell adhesion protein 1
VE-cadherin	Vascular endothelial cadherin
VEGFA	Vascular endothelial growth factor A
VWF	Von Willebrand factor
